# Exploration of the Electrophilic Reactivity of the Cytotoxic Marine Alkaloid Discorhabdin C and Subsequent Discovery of a New Dimeric C-1/N-13-Linked Discorhabdin Natural Product

**DOI:** 10.3390/md18080404

**Published:** 2020-07-31

**Authors:** Cary F. C. Lam, Melissa M. Cadelis, Brent R. Copp

**Affiliations:** School of Chemical Sciences, University of Auckland, Private Bag 92019, Auckland 1142, New Zealand; clam059@aucklanduni.ac.nz (C.F.C.L.); m.cadelis@auckland.ac.nz (M.M.C.)

**Keywords:** marine natural product, alkaloid, discorhabdin, electrophilic reactivity, discorhabdin dimer, antimalarial

## Abstract

The cytotoxic marine natural product discorhabdin C contains a 2,6-dibromo-cyclohexa-2,5-diene moiety, previously proposed to be a critical feature required for biological activity. We have determined that the dienone-ring of discorhabdin C is indeed electrophilic, reacting with thiol and amine nucleophiles, affording debrominated adducts. In the case of reaction with 1-aminopentane the product contains an unusual C-2/N-18 ring closed, double-hydrate moiety. This electrophilic reactivity also extends to proteins, with lysozyme-discorhabdin C adducts being detected by ESI mass spectrometry. These results prompted further examination of an extract of discorhabdin C-producing sponge, *Latrunculia* (*Latrunculia*) *trivetricillata*, leading to the isolation and characterisation of a new example of a C-1/N-13 linked discorhabdin dimer that shared structural similarities with the 1-aminopentane-discorhabdin C adduct. To definitively assess the influence of the dienone moiety of discorhabdin C on cytotoxicity, a semi-synthetic hydrogenation derivative was prepared, affording a didebrominated ring-closed carbinolamine that was essentially devoid of tumour cell line cytotoxicity. Antiparasitic activity was assessed for a set of 14 discorhabdin alkaloids composed of natural products and semi-synthetic derivatives. Three compounds, (-)-discorhabdin L, a dimer of discorhabdin B and the discorhabdin C hydrogenation carbinolamine, exhibited pronounced activity towards *Plasmodium falciparum* K1 (IC_50_ 30–90 nM) with acceptable to excellent selectivity (selectivity index 19–510) versus a non-malignant cell line.

## 1. Introduction

**Figure 1:**
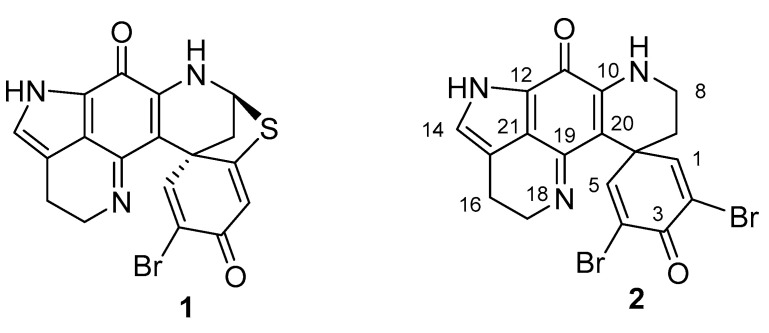
Structures of natural products (+)-discorhabdin B (**1**) and C (**2**).

Since their first report in 1987, over 40 examples of pyrroloiminoquinone alkaloids belonging to the discorhabdin/prianosin/epinardin families have been reported. Usually derived from extracts of marine sponges of the genus *Latrunculia*, these alkaloids have attracted much attention from both chemists and biologists due to their complex structures and associated biological activities [1,2]. While some of the first examples of the discorhabdins e.g., discorhabdin B (**1**) and C (**2**) (Figure 1) were structurally relatively simple [3,4], recent studies have unveiled unusual dimers [5], oligomers [6] and more complex polycyclic variants [7,8].

**Figure 2:**
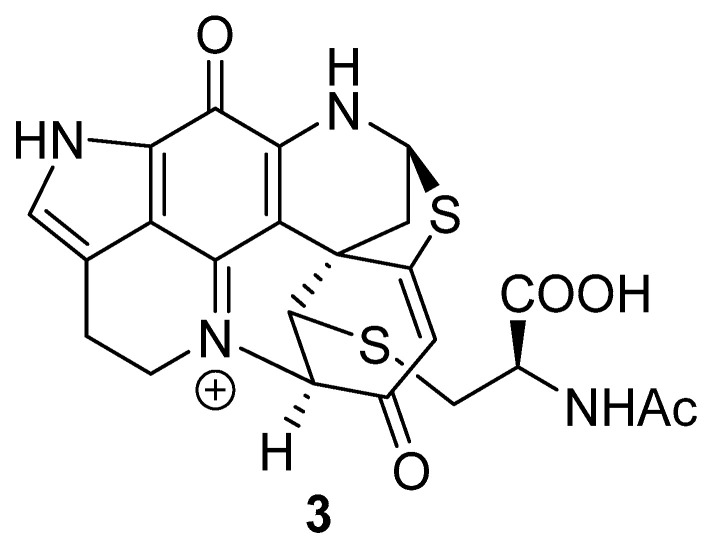
Structure of the *N*-acetyl-l-cysteine adduct of (+)-discorhabdin B (**3**).

In many cases, discorhabdin alkaloids have been reported to exhibit cytotoxicity towards tumour cell lines [1,2,3,4,6,8,9], with additional biological activities also including inhibition of protein–protein interaction associated with hypoxia-inducible factor 1α and its transcriptional coactivator p300 [10,11], antibacterial [12,13,14] and antimalarial [13] properties. The biological activities of simple dienone-containing examples of the discorhabdins has been attributed to the electrophilic properties of this moiety [2,15]. We have studied this mode of reactivity for discorhabdin B (**1**), finding that reactions with thiols including *N*-acetyl-l-cysteine affords C-1 thiol substituted C-2/N-18 ring closed analogues (e.g., **3**, Figure 2) [16] and that semi-synthetic derivatives that lack electrophilic reactivity exhibit less potent cytotoxicity. A similar conclusion was drawn from a study of *N*-13 methyl discorhabdin C (discorhabdin P) [17].

As part of our ongoing interest in the discorhabdin family of alkaloids, we now examined the electrophilic reactivity of discorhabdin C towards a series of model thiol and amine nucleophiles and the amine-rich protein lysozyme, and defined the critical role played by the dieonone moiety by preparation and biological evaluation of a hydrogenated derivative of discorhabdin C. The isolation and semi-synthetic preparation of discorhabdin alkaloids with attenuated cytotoxicity (*vide infra*) provided an opportunity to discover additional biological activities for this class of natural product—screening against a panel of parasites uncovered several examples with potent and selective activity towards *Plasmodium falciparum*. During the course of this study, a new C-1/N-13 linked discorhabdin dimer was isolated from *Latrunculia (Latrunculia) trivetricillata*. Herein we report the results of these studies.

## 2. Results

### 2.1. Reactivity towards Thiols

**Figure 3:**
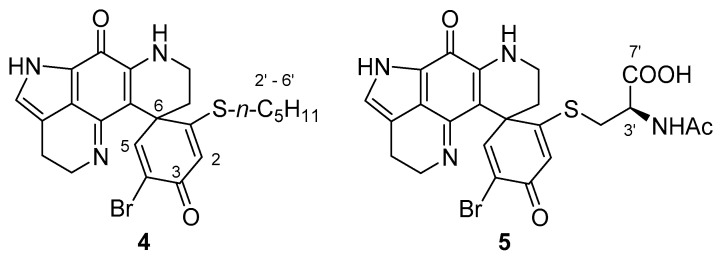
Structures of the 1-pentanethiol **4** and *N*-acetyl-l-cysteine adducts **5** of discorhabdin C.

Discorhabdin C (TFA salt) (**2**) was reacted with 1-pentanethiol (1 equiv.) in MeOH in the presence of excess triethylamine (5 equiv.) for 5 min. After this time, the dark red/black solution was directly loaded onto a C_18_ reversed-phase flash chromatography column whereby elution with H_2_O + 0.05% TFA, followed by 35–40% MeOH/H_2_O + 0.05% TFA afforded **4** (Figure 3) as a red solid in low (10%) yield. ESI-mass spectrometric data identified the product to be a mono-desbromo, mono-1-pentanethiol adduct, with HRESIMS protonated molecular ions observed at *m*/*z* 486.0838, corresponding to a formula of C_23_H_25_^79^BrN_3_O_2_S (requires 486.0845), and *m*/*z* 488.0839 (C_23_H_25_^81^BrN_3_O_2_S requires 488.0827). ^1^H and ^13^C NMR data supported the structure as being a mono-thioether adduct, with direct comparison of chemical shifts with those observed for discorhabdin C identifying the site of modification as being located in the spiro-dienone fragment of the molecule. Of note were the observation of three olefinic ^1^H singlets [δ_H_ 7.72 (1H, H-5), 7.21 (1H, H-14), 6.41 (1H, H-2)], changes observed for the discorhabdin C-core H_2_-7 and H_2_-8 resonances from triplets observed for **2** to diastereotopic multiplets in **4**, and the presence of a single unit of a 1-pentylthioether [δ_H_ 3.03–2.95 (H_2_-2′), 1.75–1.69 (H_2_-3′), 1.47–1.33 (H_2_-4′ and H_2_-5′) and 0.92 (H_3_-6′)] (Table 1). HMBC correlations observed for H-5 (δ_H_ 7.72) to δ_C_ 176 (C-3), δ_C_ 126 (C-4) and δ_C_ 46 (C-6) also identified a new ^13^C resonance (δ_C_ 170, C-1) in the spiro-dienone ring. An HMBC correlation between the pentylthioether resonances H_2_-1′ (δ_H_ 3.03–2.95) and C-1 located the thiol substituent at C-1, completing the structure of **4**.

Reaction of discorhabdin C with a second thiol nucleophile, *N*-acetyl-l-cysteine (5 equiv.) in DMF-MeOH-H_2_O mixture (1:1:0.1) and trimethylamine (7 equiv.) open to the air at room temperature for 20 min gave a change of colour from bright orange to a dark orange/brown. Purification by C_18_ reversed-phase flash column chromatography [MeOH/H_2_O (+0.05% TFA)] afforded the major product (**5**, 14%) as a 1:1 mixture of two diastereomers (Figure 3). HRESI mass spectrometry established a molecular formula of C_23_H_22_^79^BrN_4_O_5_S ([M + H]^+^ *m*/*z* 545.0482, calcd. 545.0489) consistent with the product being a mono desbromo mono-*N*-acetylcysteinyl adduct of discorhabdin C. Analysis of ^1^H and ^13^C NMR data identified the presence of a single *N*-acetylcysteine residue, while the remaining resonances were almost identical to those observed for thiopentylether analogue **4** (Table 1). The observation of doubling of resonances for H-2 (δ_H_ 6.56 and 6.58 (s)) and for *N*-acetylcysteine resonances H_2_-2′ (δ_H_ 3.57/3.54 and 3.27/3.21), H-3′ (δ_H_ 4.75 and 4.71) and *N*-Ac (δ_H_ 1.97 and 1.94) suggested the presence of diastereomers. The presence of diastereomeric products can be rationalised by considering that attack of the chiral nucleophile at either of the chemically equivalent C-1/C-5 spiro-dienone carbons of discorhabdin C would yield two products that are diastereomeric at C-6.

### 2.2. Reactivity towards Amines

The reactivity of discorhabdin C towards amine nucleophiles was investigated using 1-aminopentane and *N*α-acetyl-l-lysine. Reaction with 1-aminopentane (5 equiv.) and triethylamine (5 equiv.) in DMF for 3 h afforded a major product (31%) after purification by C_18_ reversed-phase flash chromatography. Initial ^1^H NMR analysis of the product (in D_2_O) indicated the sample contained at least two components, however over a 6 h period, the NMR spectrum simplified into one suggesting the presence of a single dominant compound. Analysis of 1D and 2D NMR data identified an *N*-substituted aminopentane fragment [δ_H_ 3.19 (2H, t, *J* = 8.0 Hz, H_2_-2′), 1.74 (2H, m, H_2_-3′), 1.34 (4H, m, H_2_-4′,H_2_-5′), 0.88 (3H, m, H_3_-6′); δ_C_ 50.5 (C-2′), 30.6 (C-4′), 27.4 (C-3′), 24.2 (C-5′), 15.7 (C-6′)] and an intact pyrroloiminoquinone moiety [δ_H_ 7.22 (H-14); δ_C_ 122.1 (C-15), 125.7 (C-21), 126.0 (C-12)] (Table 2).

**Figure 4:**
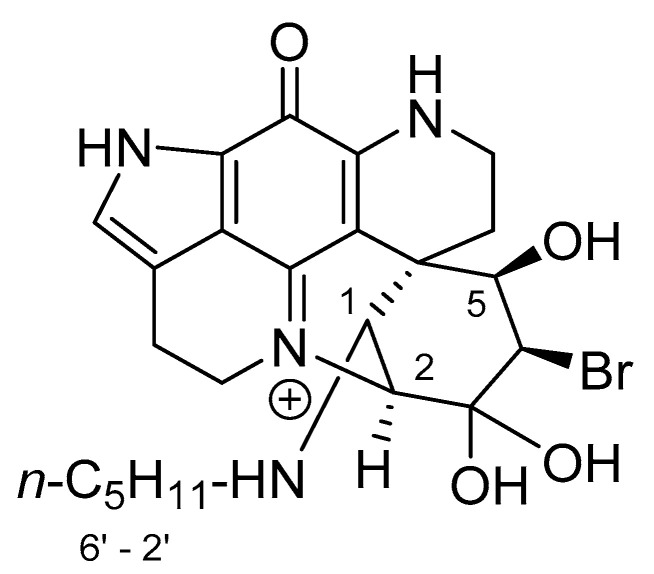
Structure of aminopentane discorhabdin C dihydrate adduct **6**.

The absence of olefinic resonances due to H-1/5 identified the site of reaction to be localised at the spiro-dienone ring, while the change in appearance of both sets of methylene resonances H_2_-16 and H_2_-17 from clearly defined triplets (in discorhabdin C) [4] to diastereotopic multiplets suggested that the alkaloid had undergone ring closure between N-18 and C-2 [18,19]. The presence of two alkyl methines [δ_H_ 4.28 (d, *J* = 2.3 Hz), δ_C_ 55.3 (CH-1); δ_H_ 4.38 (d, *J* = 2.3 Hz), δ_C_ 66.7 (CH-2)] and the observation of an HMBC correlation between H-2 and C-19 (δ_C_ 154.2) further supported N-18 to C-2 ring closure. An HMBC correlation between the pentylamine H_2_-2′ resonance (δ_H_ 3.19) and the carbon resonance at δ_C_ 55.3 located the amine substituent at C-1. This sequence of analysis left two protons (δ_H_ 4.42 and 4.15) and three ^13^C resonances (δ_C_ 98.0, 75.8 and 57.5) unassigned. HSQC correlations established the resonances to be composed of two methines (δ_H_ 4.42, δ_C_ 57.5; δ_H_ 4.15, δ_C_ 75.8) and a quaternary carbon (δ_C_ 98.0), with a weak COSY correlation between the two protons identifying them to be part of an isolated spin-system. HMBC correlations observed for H-1 (to δ_C_ 41.6 (C-6)), H-2 (to δ_C_ 41.6 (C-6), 55.6 (C-1), 57.5 (C-4) and 98.0 (C-3)) and δ_H_ 4.15 (H-5) (to δ_C_ 41.6 (C-6), 55.6 (C-1), 57.5 (C-4) and 98.0 (C-3)) established the C-1 to C-6 spiro-ring to be entirely composed of *sp*^3^-hydridised carbons, with C-3 (δ_C_ 98.0) being especially deshielded. It was interesting to note that over a period of days of being stored in D_2_O NMR solvent, the signals for H-4 (δ_H_ 4.42) and C-4 (δ_C_ 57.5) would reduce in intensity, eventually disappearing from spectra. We suspected ^2^H incorporation into **6** from the D_2_O NMR solvent, with its attendant deuterium quadrupole relaxation, spin-spin splitting and reduced Overhauser enhancement [20], being the reasons for the loss of H-4/C-4 NMR signals. Subsequent drying of the NMR sample, followed by three cycles of dissolution in H_2_O and lyophilisation then rapid re-acquisition of NMR data in either D_2_O or H_2_O:D_2_O (9:1) restored the presence of the signals. The ESI-mass spectrum of the reaction product identified a dominant ion cluster at *m*/*z* 505/507, which under high resolution analysis corresponded to a molecular formula of C_23_H_30_BrN_4_O_4_. This formula suggested **6** was composed of a mono-debrominated discorhabdin C core bearing one aminopentane moiety and two mole equivalents of H_2_O. Additional fragment ions were observed at *m*/*z* 487/489 (M-H_2_O) and *m*/*z* 469/471 (M-2H_2_O), confirming the reaction product contained two moles of H_2_O that were readily expelled under ESIMS conditions. ESIMS analysis of a sample dissolved in MeOH-H_2_O identified an additional ion cluster at *m*/*z* 519/521 (M-H_2_O+CH_3_OH) suggesting solvent exchange with methanol was also facile. The combination of NMR chemical shifts and ESIMS data identified C-3 to be present as an acetal (δ_C_ 98.0) and C-5 (δ_C_ 75.8) was a secondary alcohol. We thus concluded the structure of the product resulting from reaction of discorhabdin C with 1-aminopentane was **6** (Figure 4).

**Figure 5:**
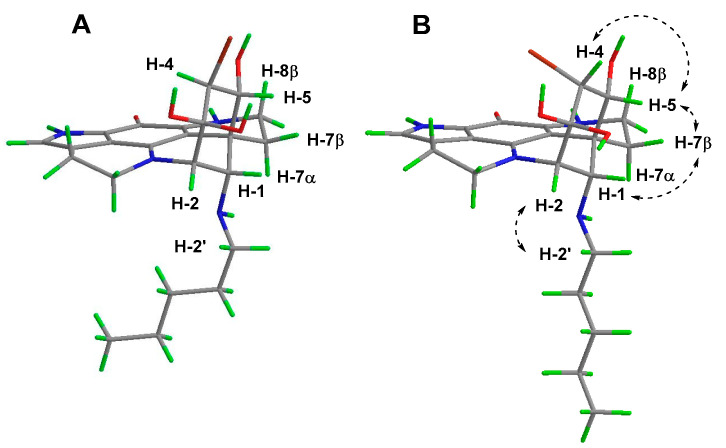
PCModel optimised dominant chair conformers of diastereomers 1S*,2S*,4S*,5R*,6R*-**6** (**A**) and 1S*,2S*,4R*,5R*,6R*-**6** (**B**) with key NOE correlations observed (**right**).

With respect to the relative stereochemistry of the product, it should be noted that the geometry resulting from ring closure between N-18 and C-2 of the discorhabdin scaffold automatically defines positions C-2 and C-6 as being 2S*,6R* (Figure 5A,B). The configuration at positions 1 and 5 were defined by the observation of NOESY correlations between each of H-1 (δ_H_ 4.28) and H-5 (δ_H_ 4.15) and the more downfield [16,19] of the diastereotopic methylene H_2_-7 protons [H-7β (δ_H_ 2.53)], and from H-5 to one of the diastereotopic H_2_-8 protons (H-8β, δ_H_ 3.62) giving configurations of 1S* and 5R*. Additional support for the *syn*-relationship between these three protons came from examination of the relative intensities of HMBC correlations observed in an experiment optimised for ^x^*J*_CH_ 8.33 Hz. Of the two diastereotopic H_2_-7 methylene protons, δ_H_ 2.53 and 1.74, only the former exhibited a three-bond correlation to C-20, supporting an antiperiplanar relationship between H-7β δ_H_ 2.53 and C-20 [19]. The H-1 resonance (δ_H_ 4.28) also exhibited a strong HMBC correlation to C-20, supporting their antiperiplanar relationship. Assigning the final stereogenic centre (C-4) made use of a combination of molecular modelling, *J* analysis and NOESY data. Conformer searching using PCModel (v 10, Serena Software) identified the dominant (chair) conformers of the two diastereomers 1S*,2S*,4S*,5R*,6R*-**6** (Figure 5A) and 1S*,2S*,4R*,5R*,6R*-**6** (Figure 5B). The observation of a small magnitude *J*_H-4/H-5_ coupling constant (*J*_HH_ = 3.4 Hz) and a NOESY correlation between the two protons was supported by model B, establishing a relative configuration of 4R*.

Reaction of discorhabdin C with *N*α-acetyl-l-lysine under the standard conditions yielded a complex mixture of products. Analysis by (+)-ESIMS identified the presence of ions at *m*/*z* 570.1353 (calcd. for C_26_H_29_^79^BrN_5_O_5_, 570.1347), 588.1490 (calcd. for C_26_H_31_^79^BrN_5_O_6_, 588.1452), 606.1546 (calcd. for C_26_H_33_^79^BrN_5_O_7_, 606.1558) and 620.1680 (calcd. for C_27_H_35_^79^BrN_5_O_7_, 620.1714) attributable to the formation of a mono-desbromo *N*α-acetyl-l-lysine adduct with various further additions of H_2_O and CH_3_OH (from MS solvent). No further characterisation of the reaction products were undertaken. In a similar manner, (+)-ESIMS analysis of the reaction of discorhabdin C with the thiol- and amine-containing nucleophile glutathione (5 equiv.) identified an extensive array of ions at *m*/*z* 689.1028/691.1013 (C_28_H_30_BrN_6_O_8_S), 725.1219/727.1196 (C_28_H_34_BrN_6_O_10_S) and 739.1402/741.1371 (C_29_H_36_BrN_6_O_10_S) consistent with the presence of *N*-/*S*-glutathionyl and H_2_O/CH_3_OH adducts.

### 2.3. Reaction with Lysozyme

The successful demonstration that discorhabdin C reacts with model amine nucleophiles prompted us to next investigate reactivity towards the lysine-rich model protein lysozyme using (+)-ESIMS to identify the presence of any reaction products [21]. Incubation of discorhabdin C with hen egg white lysozyme (HEWL) in H_2_O for 12 h gave no detectable adducts, but by day 3, two peaks representing mass additions of +382 mu (2%) and +418 mu (1%) were detected. These adducts are likely the result of the reaction of lysine residues in the protein with discorhabdin C, with the higher mass adduct corresponding to further addition of two equivalents of H_2_O, fully consistent with the 1-aminopentane adduct study.

### 2.4. A Cyclic Carbinolamine Analogue of Discorhabdin C

**Figure 6:**
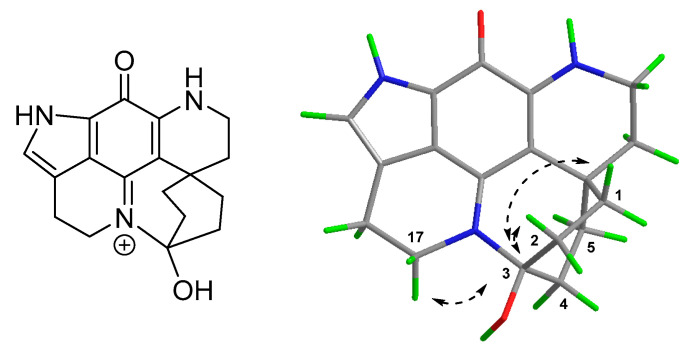
Structure of carbinolamine analogue of discorhabdin C **7** (**left**) and key HMBC correlations supporting the ring-closed structure (**right**).

Collectively these studies characterise the electrophilic reactivity of the spiro-dienone moiety of discorhabdin C, a structural feature that is known to be a factor, but not the sole determinant, for the cytotoxicity reported for this class of alkaloid [15,16,17]. To further explore the degree of essentiality of the dienone ring towards cytotoxicity, discorhabdin C was subjected to hydrogenation using Pd/C under an H_2_ atmosphere for 15 min to afford, after workup, a blue-coloured product in 71% yield. HR ESI mass spectrometry determined a formula of C_18_H_20_N_3_O_2_ ([M] + 310.1542, calcd. 310.1550) indicating didebromination and addition of two moles of H_2_. Direct comparison with ^1^H NMR data observed for discorhabdin C [4] identified the absence of H-1/5 olefinic resonances, a downfield shift for H_2_-17 (Δδ +0.38), upfield shifts for H_2_-8 (Δδ –0.24), and H_2_-7 (Δδ –0.41) and the presence of two new signals at 2.28–2.14 (m) and 1.85, with the latter two signals integrating for 4H each (Table 3). Comparison of ^13^C and HSQC 2D NMR data revealed the absence of resonances associated with the dienone moiety of **2** and the presence of new signals attributable to two chemically equivalent methylenes (δ_H_ 2.28–2.14, δ_C_ 35.9; δ_H_ 1.85, δ_C_33.8) and a deshielded ^13^C quaternary resonance at δ_C_ 92.0. HMBC correlations from H_2_-1/5 (δ_H_ 1.85), H_2_-2/4 (δ_H_ 2.28-2.14) and H_2_-17 (δ_H_ 4.15) to the resonance at δ_C_ 92.0 located the carbon at C-3 and also established ring closure between C-3 and N-18. In this manner the structure of the reaction product was concluded to be the cyclic carbinolamine **7** (Figure 6).

### 2.5. Isolation of a New Example of a C-N-Linked Discorhabdin Dimer

Having established the electrophilic reactivity of discorhabdin C, we were prompted to re-examine chromatographic fractions obtained from Latrunculia (Latrunculia) trivetricillata, the marine sponge source of discorhabdin C used in the present study. Non-purple fractions (i.e., those not containing discorhabdin C) obtained by LH-20 chromatography of the crude sponge extract were subjected to ^1^H NMR and ESIMS-directed fractionation using combinations of LH-20 and C_18_ reversed-phase column chromatography to afford **8** as a brown non-crystalline solid. Analysis of the (+)-HRESI mass spectrum of **8** identified molecular ions at m/z 879/881/883/885, with the corresponding protonated doubly charged ions at m/z 440/441/442/443 also observed. The assigned molecular formula of C_36_H_30_Br_3_N_6_O_6_ suggested the presence of a pseudo-dimer composed of two discorhabdin C-like fragments [6]. Interpretation of 1D and 2D NMR data revealed the presence of two distinct discorhabdin scaffolds, one of which was very similar to discorhabdin C (‘fragment A’), and the second (‘fragment B’) very similar to the keto-hydrate structure of aminopentane adduct **6**.

The NMR resonances observed for fragment A were similar to those observed for discorhabdin C, particularly with respect to ^1^H resonances associated with the spiro-dienone moiety [H-1/H-5 (δ_H_ 7.81 (s)), H_2_-7 (δ_H_ 2.15), and H_2_-8 (δ_H_ 3.76)] [4] (Table 4). In contrast, resonances associated H-14 (δ_H_ 6.86) and H_2_-16 (δ_H_ 2.72) of the iminoquinone core of the fragment were different (more shielded) when compared to the corresponding resonances in discorhabdin C, suggesting the presence of a substituent at the N-13 position of discorhabdin C. Investigation of the second set (fragment B) of resonances revealed a familiar structure, with ^1^H NMR resonances attributable to a ring closed (C-2′ N-18′), keto-hydrate discorhabdin-like structure, as observed for aminopentane adduct **6**. Resonances associated with the C-1′ to C-6′ ring of **8** were assigned by analysis of NMR data obtained in D_2_O and ^1^H and ^13^C NMR 1D spectra acquired in 9/1 H_2_O/D_2_O. Similar to **6**, a fragment sequence of C-1′ (δ_H_ 6.44; δc 54.1), C-2′ (δ_H_ 4.29; δc 71.2); C-3′ (δc 98.4), C-4′ (δ_H_ obscured; δc 58.6), C-5′ (δ_H_ 4.16; δc 76.0) to C-6′ (δc 42.8) was established using a combination of COSY, HSQC and HMBC NMR data. Of particular note was the unusually deshielded resonance observed for H-1′ (δ_H_ 6.44), identifying the presence of a highly electron withdrawing group at C-1′ [6]. Other proton spin-system resonances associated with fragment B were found to be almost identical to those observed for aminopentane adduct **6** including resonances for H_2_-7′ (δ_H_ 2.31, 1.07), H_2_-8′ (δ_H_ 3.89, 3.56), H-14′ (δ_H_ 7.24), H_2_-16′ (δ_H_ 3.06) and H_2_-17′ (δ_H_ 4.13, 4.04). Connectivity between the two fragments was revealed by the observation of HMBC correlations from H-1′ (δ_H_ 6.44) to both C-12 (δ_C_ 126.6) and C-14 (δ_C_ 130.1), establishing **8** to be a new example of a C-1′/N-13 linked discorhabdin dimer [6].

**Figure 7:**
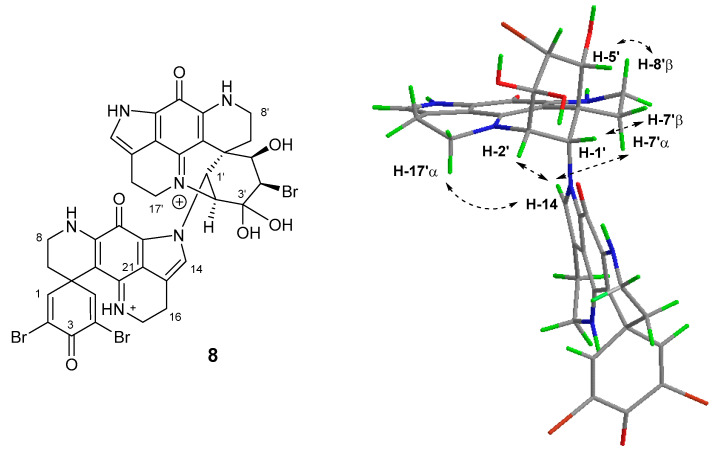
The structure of discorhabdin C dimer (**8**) (**left**) and key intra- (H-1′/H-7′β, H-5′/H-8′β) and inter-fragment (between H-14 (of fragment A) and H-17′α, H-2′ and H-7′α (of fragment B)) NOESY correlations (**right**) observed.

The relative configuration of stereogenic centres in **8** were established by interpretation of NOESY correlations, in combination with qualitative analysis of ^1^H-^13^C dihedral angles derived from the strength of HMBC NMR correlations. The first step was to again note that C-2/N-18 ring closure defines the scaffold configuration as 2′S*, 6′R*. Unfortunately only a limited number of NOESY correlations were observed for signals in the C-1 to C-6 fragment, namely H-1′ (δ_H_ 6.44) to one of the diastereotopic methylene protons at H_2_-7′ (δ_H_ 2.31), and from H-5′ (δ_H_ 4.17) to one of the diastereotopic methylene protons at H_2_-8′ (δ_H_ 3.56). Differentiating between the H_2_-7′ methylene protons (δ_H_ 2.31, 1.07) as either α (axial) or β (equatorial) was achieved by examination of the intensity of 3-bond ^1^H-^13^C HMBC correlations, in a similar manner to that used earlier for adduct **6**. Of the methylene two protons, only one of them (δ_H_ 1.07) exhibited a strong correlation to C-5′ (δ_C_ 76.0) in an HMBC experiment optimised to ^x^J_CH_ 8.33 Hz. As only the α-faced (axial) proton is in an antiperiplanar geometry with C-5′ (PCModel conformer search and optimised geometry, Figure 7), the methylene resonance at δ_H_ 1.07 was attributed to H-7′α, leaving the remaining H-7′ resonance at δ_H_ 2.31 as the 7’β (equatorial) proton [19]. Thus, the observation of a NOESY correlation from H-1′ to H-7′β defined configuration at C-1′ as S*. No useful NOESY correlations were observed for H-5′, however the similarity of chemical shifts and J_HH_ coupling constants for H-2/H-4 and H-5 of both **6** and **8** gave us confidence to assign similar relative configuration to **8**, i.e., 1S*,2S*,4R*,5R*,6R* (Figure 7). Strong inter-fragment NOESY correlations were observed between H-14 in fragment A and H-2″ (δ_H_ 4.28), H-7′α (δ_H_ 1.07) and H_2_-17′ (H-17′α, δ_H_ 4.00) in fragment B, providing further evidence for the C-1α (axial) orientation of the N-13 substituted discorhabdin C fragment A (Figure 7). No specific rotation nor circular dichroism Cotton Effects were observed at any wavelengths, identifying that **8** had been isolated as the racemate.

To examine the possibility of this natural product being an artefact produced during isolation, samples of free base discorhabdin C were left to stir in the presence of TEA and water. Reaction was monitored by HPLC at 12 h intervals, with no significant change observed. The reaction was halted after 48 h, which yielded small quantities of discorhabdin C and a decomposed mixture, analysis of which did not indicate the presence of any dimer-related products.

### 2.6. Assessing Biological Activities

Discorhabdin C (**2**) and analogues **4**, **6** and **7** were evaluated for cytotoxicity towards a panel of human tumour cell lines as part of the Developmental Therapeutics Program of the National Cancer Institute. Unfortunately, due to compound instability, dimer **8** was not submitted for testing. The thiopentanyl analogue **4** was found to be approximately an order of magnitude less active than the parent alkaloid discorhabdin C (Table 5). Of note was that analogues **6** (NSC 791238) and **7** (NSC 791237), both of which lack any dienone functionality, were deemed inactive in preliminary single dose (10 μM) testing, with tumour cell mean growth values of 100.67% (delta 33.70, range 63.59) and 99.57% (delta 37.53, range 59.64), respectively. Neither compound was progressed to determination of GI_50_/TGI/LC_50_ values. The results for these latter two semi-synthetic derivatives provides direct support for the critical role played by the spiro-dienone ring in the cytotoxic properties of discorhabdin C.

**Figure 8:**
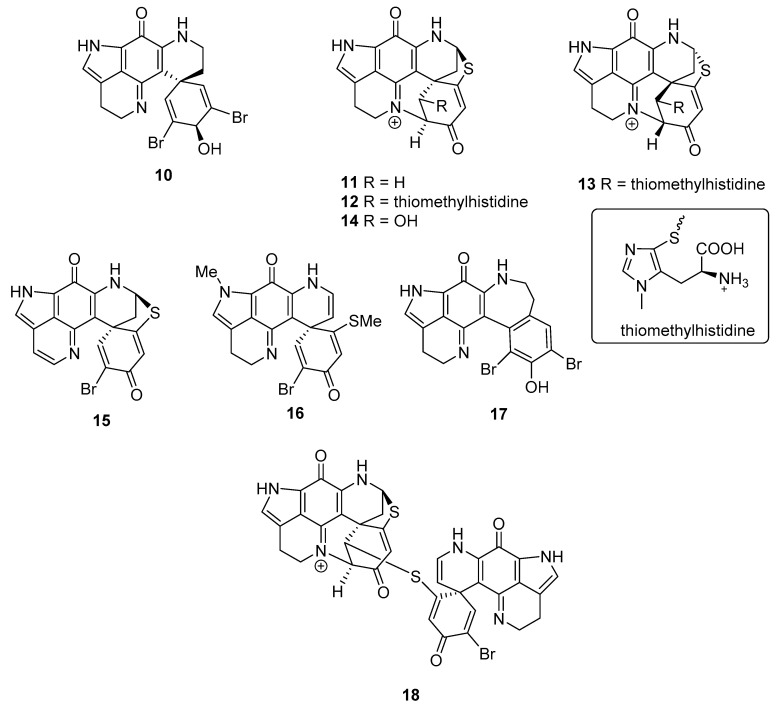
Structures of additional discorhabdin natural product and semi-synthetic analogues **9**–**17** that were evaluated for antiparasitic and cytotoxic properties.

Natural products containing the iminoquinone scaffold have been reported to exhibit antimalarial activity, including discorhabdin C, which was active (*Plasmodium falciparum* (*Pf*) D6 clone IC_50_ 2.8 μM, *Pf* W2 clone IC_50_ 2.0 μM) but with negligible selectivity (Vero cell line IC_50_ 2.8 μM), and 3-dihydrodiscorhabdin C (**9**) (Figure 8) with strong and selective anti-*Pf* activity (D6 IC_50_ 0.17 μM, *Pf* W2 IC_50_ 0.13 μM, Vero IC_50_ 9.8 μM) [13]. The structurally-simpler alkaloids tsitsikammamine C and makaluvamines J, G and L, each of which lack a spiro-dienone moiety, exhibited pronounced in vitro activity towards *Pf* clones 3D7 and Dd2 (IC_50_ 13–40 nM), with only negligible cytotoxicity towards the HEK293 cell line (IC_50_ 1.2–3.6 μM) [22]. Together these results prompted us to evaluate the antiparasitic activities of a series of discorhabdin natural products and semi-synthetic analogues that we have previously shown to exhibit modest or minimal tumour cell line cytotoxicity. Included in this study were (+)-discorhabdin B **1**, discorhabdin C **2**, **6**–**8**, 3-dihydrodiscorhabdin C **9** [15], (+)-discorhabdin D **10**, (-)-discorhabdin H **11**, (+)-discorhabdin H_2_ **12** [19] (-)-discorhabdin L **13**, discorhabdin Q **14**, discorhabdin U **15**, semi-synthetic dienone-phenol **16** [23] and discorhabdin B dimer **17** [5,16] (Figure 8).

The set of compounds were screened against a panel of four parasitic protozoa: *Trypanosoma brucei rhodesiense*, *Trypanosoma cruzi*, *Leishmania donovani* and *Plasmodium falciparum* K1 dual drug-resistant strain. Initial testing was part of a larger library medium-throughput-screen, using two set doses (4.81 and 0.81 μg/mL). Compounds that exhibited some degree of selectivity (i.e., were not strongly active against three or four parasites which was considered a sign of general toxicity) were then evaluated for IC_50_ against the parasites and for cytotoxicity towards the L6 rat myoblast cell line (Table 6).

All compounds exhibited some degree of antimalarial activity, with natural product (-)-discorhabdin L (**13**) and semi-synthetic analogues **7** and **17** being particularly potent (IC_50_ 30, 90 and 80 nM, respectively) and selective versus the L6 mammalian cell line (SI 37, 19 and 510, respectively). Additionally, also of note were the sub-micromolar activities of **8**, **13** and **17** towards *Trypanosoma brucei rhodesiense*. In the case of discorhabdin B dimer **17**, it is interesting to contrast the low cytotoxicity towards the non-malignant rat skeletal myoblast cell line L6 (IC_50_ 41 μM) (Table 6), compared to the recently reported strong cytotoxicity observed for the same compound towards a human colon tumour cell line HCT-116 (IC_50_ 0.16 μM) and a non-malignant human keratinocyte cell line (IC_50_ 0.56 μM) [5].

## 3. Discussion

**Figure 9:**
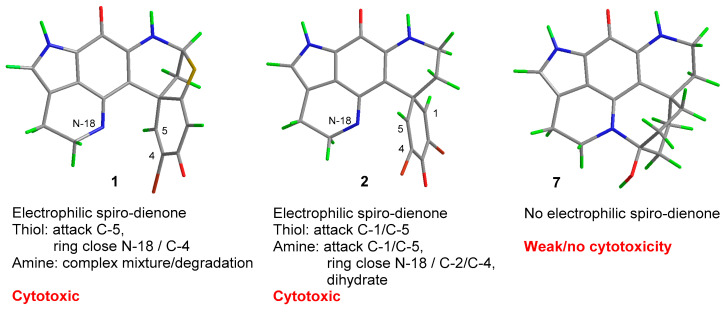
Summary of electrophilic reactivity/cytotoxicity observed for discorhabdin B (**1**) [16] and discorhabdin C (**2**) and cyclic carbinolamine derivative (**7**) (present study).

In an effort to understand the potential role played by the spiro-(di)enone moiety in the cytotoxicity of discorhabdin alkaloids, we have previously investigated the electrophilic reactivity of discorhabdin B towards biomimetic nucleophiles [16] and prepared semi-synthetic analogues of discorhabdin P [17]. Collectively these studies concluded that electrophilic reactivity was a major, though not exclusive, source of alkaloid cytotoxicity. In the present study, we examined the reactivity of discorhabdin C, using similar methodology of determining reactivity toward nucleophiles and the cytotoxic evaluation of a semi-synthetic analogue that lacks the dibromo-dienone ‘warhead’ [23]. Interestingly when compared with discorhabdin B reactivity, the results observed for discorhabdin C are quite distinct. While both compounds reacted with thiol nucleophiles, the structures of the products were noticeably different with (+)-discorhabdin B (**1**) yielding a C-1 thiol-substituted compound that also embodied a C-2/N-18 ring closure (e.g., **3**) with discorhabdin C (**2**) reacting to afford thiol-substituted spiro-dienones **4** and **5** (Figure 9). Presumably the thioether group present in the natural product **1**, but absent in **2**, provides enough distortion to the geometry of the spiro-dienone ring to facilitate ring closure to yield **3**. In direct contrast however were the results of reaction of **1** and **2** with amine-containing nucleophiles. While the reaction of (+)-discorhabdin B with amine nucleophiles yielded intractable mixtures and natural product decomposition [16], discorhabdin C afforded a product from reaction with 1-aminopentane that embodied C-1 attack and C-2/N-18 ring closure. From such a sequence of steps, one would expect the product to contain an enone moiety: in the case of **6** however, the end product, when characterised in aqueous solvent, was the result of further addition of two mole equivalents of water. These results prompted us to re-examine extracts of the discorhabdin C-producing sponge (*Latrunculia* (*Latrunculia*) *trivetricillata*) used in the current study, leading to the isolation and characterisation of a new discorhabdin dimer **8**. The structure is closely related to discorhabdin B oligomers recently reported from a deep sea collection of *Latrunculia biformis* [6] with all examples featuring a C-1/N-13 linkage between discorhabdin fragments. It is interesting to note that while the di- and tri-discorhabdin structures contained a β-configuration (equatorial) C-1/N-13 connection and the trimer exhibited potent cytotoxicity towards the HCT-116 cell line (IC_50_ 0.31 μM), dimer **8** contained an α-configuration (axial) linkage and was only weakly cytotoxic to the L6 rat myoblast cell line (IC_50_ 2.1 μM).

One of our goals of seeking clarity on the contribution of the spiro-dienone ring to the observed biological activity (cytotoxicity) of discorhabdin C was achieved by semi-synthesis of carbinolamine **7**. Pd/C/H_2_ hydrogenation of discorhabdin C was expected to yield a desbromo-spiro-cyclohexanone product which was anticipated to be incapable of electrophilic reactivity and hence to exhibit attenuated cytotoxicity. Indeed, the product, which was characterised as the ring-closed C-3/N-18 carbinolamine, was found to be devoid of antitumour activity when tested against the human 60 cell-line panel at the NCI (Figure 9). Non- or poorly cytotoxic discorhabdin analogues were also shown to exhibit favourable levels of antimalarial activity, opening a further avenue for investigation of this renowned class of marine natural products.

## 4. Materials and Methods

### 4.1. General Experimental Procedures

Infrared spectra were recorded on a Perkin-Elmer spectrometer 100 Fourier Transform infrared spectrometer equipped with a universal ATR accessory (PerkinElmer, Boston, MA, USA). Mass spectra were acquired on a Bruker micrOTOF Q II spectrometer (Bruker^®^, Billerica, MA, USA). NMR spectra were recorded at 298 K on Bruker AVANCE 400, 500 and 600 spectrometers (Bruker^®^, Billerica, MA, USA) using standard pulse sequences. Proto-deutero solvent signals were used as internal references (CD_3_OD: δ_H_ 3.30, δ_C_ 49.00) or in the case of D_2_O or H_2_O/D_2_O mixtures, dioxane was used as an external reference (δ_H_ 3.75; δ_C_ 69.3). For ^1^H NMR, the data are quoted as position (δ), relative integral, multiplicity (s = singlet, d = doublet, t = triplet, q = quartet, m = multiplet, br = broad), coupling constant (*J*, Hz), and assignment to the atom. The ^13^C NMR data are quoted as position (δ), and assignment to the atom. The original spectra of the relative compounds could be found in Supplementary Materials. Flash column chromatography was carried out using Merck (Manukau, Auckland) LiChroPrep C_18_ reversed-phase (40–63 or 25–40 µm) solid support. Thin layer chromatography was conducted on Merck DC-plastikfolien Kieselgel 60 F254 or on Merck DC Kieselgel 60 RP-18 F254S plates. Analytical reversed-phase HPLC was run on a Waters 600 HPLC photodiode array system (Milford, MA, USA) using an Alltech C_8_ column (3 μm Econosphere Rocket, 7 × 33 mm) (Grace, Columbia, MA, USA) and eluting with a linear gradient of H_2_O (0.05% TFA) to MeCN over 13.5 min at 2 mL/min. All solvents used were of analytical grade or better and/or purified according to standard procedures. Chemical reagents used were purchased from standard chemical suppliers and used as purchased. Samples of discorhabdin C used in this study were isolated from specimens of *Latrunculia* (*Latrunculia*) *trivetricillata* (MNP 6116) collected from the Three Kings Islands, New Zealand using protocols previously reported [19]. Antitumour cell lines were sourced from the DCTD Tumor repository, National Cancer Institute at Frederick, Maryland USA. Strains of *T. B. rhodesiense*, *T. cruzi*, *L. donovani* and *P. falciparum* were obtained from Swiss Tropical and Public Health Institute. 

### 4.2. Semi-Synthesis

#### 4.2.1. Thiopentanyl-Discorhabdin C Adduct (**4**)

To a solution of discorhabdin C trifluoroacetate salt (0.045 g, 0.1 mmol) in MeOH (1 mL) was added triethylamine (0.049 g, 0.5 mmol) followed by 1-pentanethiol (0.010 g, 0.1 mmol). The reaction mixture was stirred for 5 min before being directly loading onto a reversed-phase C_18_ chromatography column and washed with three column volumes of H_2_O (+0.05% TFA). Elution with 35–40% aq. MeOH (+0.05% TFA) afforded **4** as a red-purple non-crystalline trifluoroacetate salt (5.0 mg, 10%). R_f_ (CH_2_Cl_2_/MeOH, 9:1) 0.57; IR (ATR) v_max_ 3381, 1679, 1204, 1136 cm^−1^; ^1^H NMR (CD_3_OD, 500 MHz) and ^13^C NMR (CD_3_OD, 125 MHz) Table 1; (+)-HRESIMS [M+H]^+^ *m*/*z* 486.0838 (calcd. for C_23_H_25_^79^BrN_3_O_2_S, 486.0845), 488.0839 (calcd. for C_23_H_25_^81^BrN_3_O_2_S, 488.0827).

#### 4.2.2. *N*-Acetyl-l-Cysteinyl Discorhabdin C Adduct (**5**)

*N*-Acetyl-l-cysteine (26 mg, 0.16 mmol) was dissolved in DMF (0.5 mL), MeOH (1 mL) and water (0.1 mL), followed by addition of TEA (23 µL, 0.16 mmol). Free base discorhabdin C (15.0 mg, 32.0 µmol) was dissolved in DMF (0.5 mL), followed by addition of TEA (9 µL, 64 µmol) and the *N*-acetyl-l-cysteine mixture. The reaction mixture was stirred in air for 30 min before loading the reaction mixture directly onto a reversed-phase C_18_ chromatography column and washed with three column volumes of water (0.05% TFA). Elution with 10% MeOH (0.05% TFA) yielded a brown fraction which was further purified using C_18_ (25–40 µm) column chromatography eluting with 10% MeOH in H_2_O (0.05% TFA) to afford a mixture of diastereomers (1:1) **5** (3.0 mg, 14%) as purple non-crystalline trifluoroacetate salts. R_T_ 4.48 min; IR (ATR) 3267, 1673, 1545, 1200 cm^−1^; ^1^H NMR (CD_3_OD, 600 MHz) and ^13^C NMR (CD_3_OD, 100 MHz) Table 1; (+)-HRESIMS *m*/*z* [M+H]^+^ 545.0482 (calcd. for C_23_H_22_^79^BrN_4_O_5_S, 545.0489), 547.0468 (calcd. for C_23_H_22_^81^Br_2_N_4_O_5_S, 547.0469). Resonances for C-3′ were obtained indirectly from HSQC NMR data.

#### 4.2.3. Aminopentane Discorhabdin C Dihydrate Adduct (**6**)

A solution of 1-aminopentane (12 µL, 0.12 mmol), DMF (2 mL) and TEA (18 µL, 0.13 mmol) was added to discorhabdin C trifluoroacetate salt (12.0 mg, 0.021 mmol). The reaction mixture was stirred in air for 3 h before being loaded directly onto a reversed-phase C_18_ chromatography column and washing with three column volumes of water (0.05% TFA). The first purple fraction eluted at 10% MeOH (0.05% TFA) was collected and dried in vacuo, before re-dissolving in H_2_O. After 12 h, the sample was dried in vacuo to yield compound **6** (4.0 mg, 31%) as a purple non-crystalline trifluoroacetate salt. R_T_ 3.10 min; IR (ATR) 3195, 1669, 1541, 1198, 1135 cm^−1^; ^1^H NMR (D_2_O, 400 MHz) and ^13^C NMR (D_2_O, 100 MHz) Table 2; (+)-HRESIMS *m*/*z* [M]^+^ 469.1220 (calcd. for C_23_H_26_^79^BrN_4_O_2_, 469.1234), 471.1205 (calcd. for C_23_H_26_^81^BrN_4_O_2_, 471.1214), 487.1313 (calcd. for C_23_H_28_^79^BrN_4_O_3_, 487.1339), 489.1307 (calcd. for C_23_H_28_^81^BrN_4_O_3_, 489.1320), 505.1438 (calcd. for C_23_H_30_^79^BrN_4_O_4_, 505.1445), 507.1419 (calcd. for C_23_H_30_^81^BrN_4_O_4_, 507.1426), 519.1594 (calcd. for C_24_H_32_^79^BrN_4_O_4_, 519.1601), 521.1568 (calcd. for C_24_H_32_^81^BrN_4_O_4_, 521.1582).

#### 4.2.4. Discorhabdin C Hydrogenation Product (**7**)

Discorhabdin C (**2**) as the trifluoroacetate salt (8.00 mg, 13.9 µmol) was dissolved in dry MeOH (4 mL), followed by addition of a spatula tip of Pd/C (10%). The reaction mixture was purged under H_2_ and stirred at r.t. for 15 min. The crude reaction product was filtered through a cotton wool plug, and the solvent removed in vacuo. Purification using reversed-phase C_18_ flash column chromatography (10% to 25% MeOH/H_2_O + 0.05% TFA) afforded **7** as a blue non-crystalline trifluoroacetate salt (4.20 mg, 71%). R_f_ (30% (10% aq. HCl) 0.68, 70% MeOH); IR (ATR) 3390, 3239, 1671, 1194, 1173, 1126 cm^−1^; ^1^H NMR (CD_3_OD, 400 MHz) and ^13^C NMR (CD_3_OD, 100 MHz) Table 3; (+)-HRESIMS *m*/*z* [M]^+^ 310.1542 (calcd. for C_18_H_20_N_3_O_2_, 310.1550).

### 4.3. Isolation and Purification of Discorhabdin C (***2***) and Dimer (***8***)

Freeze dried *Latrunculia* (*Latrunculia*) *trivetricillata* sponge material (7.3 g) was extracted with MeOH (4 × 250 mL). The solvent was filtered and then removed in vacuo to give a purple/brown crude extract (1.62 g). The crude extract was subjected to Sephadex LH-20 (MeOH (0.05% TFA)) column chromatography to afford brown- and purple-coloured fractions. The brown fraction was further purified with Sephadex LH-20 (MeOH (0.05% TFA)), followed by reversed-phase C_18_ column chromatography (10% MeOH/H_2_O (0.05% TFA)) to give dimer **8** as a brown non-crystalline trifluoroacetate salt (9.5 mg, 0.13%, dry weight). Further purification of the purple fraction using reversed-phase C_18_ column chromatography (10% MeOH/H_2_O (0.05% TFA)) afforded discorhabdin C (**2**) (155.5 mg, 2.13% dry weight), spectroscopic data for which matched literature values [4].

Dimer **8**: R_T_ 5.57 min; IR (ATR) 3261, 1676, 1541, 1202, 1134 cm^−1^; UV-Vis (MeOH) λ_max_ (log ε) 250 (4.49), 366 (4.15), 396 sh (4.08), 545 (3.22) nm; ^1^H NMR (D_2_O, 400 MHz) and ^13^C NMR (90% H_2_O + 10% D_2_O, 100 MHz) Table 4; (+)-HRESIMS m/z [M]^+^ 878.9782 (calcd. for C_36_H_30_^79^Br_3_N_6_O_6_, 878.9771), 880.9748 (calcd. for C_36_H_30_^79^Br_2_^81^BrN_6_O_6_, 880.9753), 882.9741 (calcd. for C_36_H_30_^79^Br ^81^Br_2_N_6_O_6_, 882.9732), 884.9692 (calcd. for C_36_H_30_^81^Br_3_N_6_O_6_, 884.9712). The NMR signal for C-4′ were observed in a spectrum acquired in H_2_O/D_2_O 9:1. The signal for H-4′ was obscured by NMR solvent.

### 4.4. Biological Assays

#### 4.4.1. Antitumour Cytotoxicity

Protocols of testing at the DTP, NCI have been described elsewhere [24,25].

#### 4.4.2. Antiparasitic and L6 Cytotoxicity Assays

Detailed protocols for these assays have been described elsewhere [26].

## Figures and Tables

**Table 1:** ^1^H and ^13^C NMR data for compounds **4** and **5** in CD_3_OD (δ in ppm).

No.	4		No.	5	
	δ_H_ (Mult., *J* in Hz) ^a^	δ_C_^b^		δ_H_ (Mult., *J* in Hz) ^c^	δ_C_^d^
1	-	169.7		-	167.5
2	6.41 (s)	121.7		6.56/6.58 (s)	122.2/122.4
3	-	176.2		-	176.2
4	-	125.9		-	126.0
5	7.72 (s)	152.1		7.71 (s)	152.2
6		47.3			47.3
7	2.39–2.33 (m)	39.2/39.4		2.34 (m)	39.2/39.4
	2.16–2.12 (m)			2.13 (m)	
8	3.92–3.87 (m)	39.2/39.4		3.88 (m)	39.2/39.4
	3.73–3.67 (m)			3.69 (m)	
10	-	154.1		-	154.2
11	-	166.4		-	166.4
12	-	125.3		-	125.4
14	7.21 (s)	127.9		7.20 (s)	127.9
15		121.4		-	121.5
16	2.95–2.83 (m)	19.6		2.89 (m)	19.5
17	3.79 (t, 7.4)	45.2		3.84–3.76 (m)	45.3/45.4
19	-	156.2		-	156.1
20	-	94.8		-	94.3
21	-	124.6		-	124.6
	**S-*n*-C_5_H_11_**			**S-NAc-cysteine**	
2’	3.03–2.95 (m)	32.2/32.5	2′	3.54/3.57 (m)	33.8/34.2
3’	1.75–1.69 (m)	28.4		3.21/3.27 (m)	
4’	1.47–1.33 (m)	32.2/32.5	3′	4.71/4.75 (m)	51.5/52.0
5’	1.47–1.33 (m)	23.2	5′	-	173.5
6’	0.92 (t, 7.2)	14.2	6′	1.94/1.97 (s)	22.4
			7′	-	168.1

^a^ 500 MHz for ^1^H NMR; ^b^ 125 MHz for ^13^C NMR; ^c^ 600 MHz for ^1^H NMR; ^d^ 100 MHz for ^13^C NMR.

**Table 2:** ^1^H (400 MHz) and ^13^C (100 MHz) NMR data for compound **6** in D_2_O (δ in ppm) ^a^.

No.	δ_H_ (Mult., *J* in Hz)	δ_C_
1	4.28 (d, 2.3)	55.3
2	4.38 (d, 2.3)	66.7
3	-	98.0
4	4.42 (d, 3.4)	57.5
5	4.15 (br m)	75.8
6		41.7
7	2.53 (d, 14.0)	25.0
	1.74 (m)	
8	3.90 (dd, 14.0, 4.3)	40.5
	3.62 (m)	
10	-	155.2
11	-	168.2
12	-	126.0
14	7.22 (s)	131.0
15		122.1
16	3.04 (m)	22.2
17	4.22 (m)	55.4
	4.06 (m)	
19	-	154.2
20	-	92.0
21	-	125.7
2’	3.19 (t, 8.0)	50.5
3’	1.74 (m)	27.4
4’	1.34 (m)	30.6
5’	1.34 (m)	24.2
6’	0.88 (m)	15.7

^a^ External reference to dioxane (δ_H_ 3.75; δ_C_ 69.3).

**Table 3:** ^1^H (400 MHz) and ^13^C (100 MHz) NMR data for compound **7** in CD_3_OD (δ in ppm).

No.	δ_H_ (Mult., *J* in Hz)	δ_C_
1	1.85 (t, 7.2)	33.8
2	2.28–2.14 (m)	35.9
3	-	92.0
4	2.28–2.14 (m)	35.9
5	1.85 (t, 7.2)	33.8
6	-	35.1
7	1.68 (t, 5.5)	36.2
8	3.46 (t, 5.5)	39.1
10	-	151.4
11	-	167.7
12	-	124.8
14	7.10 (s)	126.5
15	-	121.2
16	2.91 (t, 7.1)	21.1
17	4.15 (t, 7.1)	45.8
19	-	157.1
20	-	106.4
21	-	125.4

**Table 4:** ^1^H (400 MHz) and ^13^C (100 MHz) NMR data for compound **8** (δ in ppm).

No.	δ_H_ (Mult., *J* in Hz) ^a^	δ_C_^b^	No.	δ_H_ (Mult., *J* in Hz) ^a^	δ_C_^b^
1	7.81 (s)	156.0	1’	6.44 (d, 2.0)	54.1
2	-	124.9	2’	4.29 (d, 2.0)	71.2
3	-	177.0	3’	-	98.4
4	-	124.9	4’	Obscured	58.6
5	7.81 (s)	156.0	5’	4.16 (br m)	76.0
6	-	47.7	6’	-	42.8
7	2.15 (t, 5.5)	35.3	7’	2.31 (dd, 13.5, 2.4)	24.3
8	3.76 (m)	41.1		1.07 (td, 13.5, 5.4)	
10	-	154.9	8’	3.89 (m)	40.6
11	-	169.6		3.56 (m)	
12	-	126.6	10’	-	154.9
14	6.86 (s)	130.1	11’	-	168.8
15	-	124.5	12’	-	125.5/126.2
16	2.72 (m)	20.7	14’	7.24 (s)	130.8
17	3.89 (m)	46.6	15’	-	122.3
19	-	156.5	16’	3.06 (m)	22.2
20	-	93.7/94.0	17’	4.13 (m)	55.4
21	-	126.6		4.04 (m)	
			19’	-	154.2
			20’	-	93.7/94.0
			21’	-	125.5/126.2

^a^ D_2_O; ^b^ 90% H_2_O + 10% D_2_O. Both sets of data externally reference to dioxane (δ_H_ 3.75; δ_C_ 69.3).

**Table 5:** In vitro antitumour activities (μM) of discorhabdin C (**2**) and thiopentanyl analogue **4.**

Compound (NSC) ^1^	GI_50_^2^	TGI ^2^	LC_50_^2^
**2** (626162)	0.13	0.36	2.3
**4** (789237)	1.4	3.9	10.5

^1^ NSC number is the NCI reference number for each compound. Search for this number at http://dtp.cancer.gov to view complete information of all in vitro assay profiles; ^2^ GI_50_ (50% growth inhibition), TGI (total growth inhibition) and LC_50_ (50% cell kill) data are averaged calculated mean micro-molar values obtained from two experiments at the NCI.

**Table 6:** Anti-protozoal and cytotoxic activities of discorhabdin natural products and semisynthetic analogues **1**, **2**, **6**–**17**.

Compd.	*T. B. Rhod.* ^b^	*T. Cruzi* ^c^	*L. Don.* ^d^	*P. Falc.* K1 ^e^	L6 ^f^	SI *Pf* ^g^
**1**	99% (1.5)	100% (1.5)	57% (1.5)	100% (1.5)	n.t. ^h^	-
**2**	99% (1.4)	100% (1.4)	47% (1.4)	100% (1.4)	n.t.	-
**6**	n.t.	n.t.	n.t.	0.25	0.81	3.2
**7**	n.t.	n.t.	n.t.	0.09	1.7	19
**8**	0.71	11.1	18.7	6.4	2.1	0.3
**9**	n.t.	n.t.	n.t.	0.40	0.11	0.3
**10**	99% (1.8)	100% (1.8)	75% (1.8)	100% (1.8)	n.t.	-
**11**	1.4	29	64	1.6	9.1	5.7
**12**	2.3	63	94	12	17	1.4
**13**	0.40	3.6	7.2	0.03	1.1	37
**14**	1.3	9% (12)	20% (1.9)	20% (1.9)	11	-
**15**	100% (1.5)	100% (1.5)	97% (1.5)	100% (1.5)	n.t.	-
**16**	n.t.	n.t.	n.t.	1.7	6.1	3.6
**17**	0.33	0% (0.8)	29% (0.8)	0.08	41	510

^a^ IC_50_ values in μM, or % inhibition at the μM dose stated. All data is the mean value from duplicate assays; ^b^ *Trypanosoma brucei rhodesiense* (positive control melarsoprol, IC_50_ 0.01 μM); ^c^ *Trypanosoma cruzi* (positive control benznidazole, IC_50_ 1.35 μM); ^d^ *Leishmania donovani* (positive control miltefosine, IC_50_ 0.52 μM); ^e^ *Plasmodium falciparum*, K1 strain (positive control chloroquine, IC_50_ 0.20 μM); ^f^ L6 rat skeletal myoblast cell line for cytotoxicity (positive control podophyllotoxin, IC_50_ 0.01 μM); ^g^ Antiplasmodial selectivity index = L6 IC_50_/P f. IC_50_; ^h^ Not tested.

## Supplementary Materials

The following are available online at https://www.mdpi.com/1660-3397/18/8/404/s1, Figure S1–S29: NMR spectra of compounds **4**–**8**.
