# Supplementary material for: Exploration of the Electrophilic Reactivity of the Cytotoxic Marine Alkaloid Discorhabdin C and Subsequent Discovery of a New Dimeric C-1/N-13-Linked Discorhabdin Natural Product
Source: Mar Drugs. 2020 Jul 31;18(8):404. doi: 10.3390/md18080404 (PMC7460216; doi:10.3390/md18080404)
Supplement: Supplementary file 1 [file marinedrugs-18-00404-s001.pdf]

SUPPLEMENTARY MATERIAL

Exploration of the electrophilic reactivity of the cytotoxic marine alkaloid discorhabdin C and subsequent discovery of a new dimeric C-1/N-13-linked discorhabdin natural product

Cary F.C. Lam[1], Melissa M. Cadelis[1] and Brent R. Copp[1*]

[1] School of Chemical Sciences, University of Auckland, Private Bag 92019, Auckland 1142, Aotearoa New Zealand; clam059@aucklanduni.ac.nz; m.cadelis@auckland.ac.nz; b.copp@auckland.ac.nz

* Correspondence: b.copp@auckland.ac.nz

Contents:

Figure S1. ^{1}H NMR spectrum of compound **4** (TFA salt, CD_3OD, 500 MHz).
Figure S2. ^{13}C NMR spectrum of compound **4** (TFA salt, CD_3OD, 125 MHz).
Figure S3. COSY spectrum of compound **4** (TFA salt, CD_3OD, 500 MHz).
Figure S4. HSQC spectrum of compound **4** (TFA salt, CD_3OD, 500 MHz).
Figure S5. HMBC spectrum of compound **4** (TFA salt, CD_3OD, 500 MHz).
Figure S6. NOESY spectrum of compound **4** (600 ms mixing time, TFA salt, CD_3OD, 500 MHz).

Figure S7. ^{1}H NMR spectrum of compound **5** (TFA salt, CD_3OD, 600 MHz).
Figure S8. ^{13}C NMR spectrum of compound **5** (TFA salt, CD_3OD, 100 MHz).
Figure S9. COSY spectrum of compound **5** (TFA salt, CD_3OD, 400 MHz).
Figure S10. HSQC spectrum of compound **5** (TFA salt, CD_3OD, 600 MHz).
Figure S11. HMBC spectrum of compound **5** (TFA salt, CD_3OD, 600 MHz).

Figure S12. ^{1}H NMR spectrum of compound **6** (TFA salt, D_2O, 400 MHz).
Figure S13. ^{13}C NMR spectrum of compound **6** (TFA salt, D_2O, 100 MHz).
Figure S14. COSY spectrum of compound **6** (TFA salt, D_2O, 400 MHz).
Figure S15. HSQC spectrum of compound **6** (TFA salt, D_2O, 400 MHz).
Figure S16. HMBC spectrum of compound **6** (TFA salt, D_2O, 400 MHz).
Figure S17. NOESY spectrum of compound **6** (600 ms mixing time, TFA salt, D_2O, 400 MHz).

Figure S18. ^{1}H NMR spectrum of compound **7** (TFA salt, CD_3OD, 400 MHz).
Figure S19. ^{13}C NMR spectrum of compound **7** (TFA salt, CD_3OD, 100 MHz).
Figure S20. COSY spectrum of compound **7** (TFA salt, CD_3OD, 400 MHz).
Figure S21. HSQC spectrum of compound **7** (TFA salt, CD_3OD, 400 MHz).
Figure S22. HMBC spectrum of compound **7** (TFA salt, CD_3OD, 400 MHz).
Figure S23. NOESY spectrum of compound **7** (600 ms mixing time, TFA salt, CD_3OD, 400 MHz).

Figure S24. ^{1}H NMR spectrum of compound **8** (TFA salt, D_2O, 400 MHz).
Figure S25. ^{13}C NMR spectrum of compound **8** (TFA salt, 90% H_2O : 10% D_2O, 100 MHz).
Figure S26. COSY spectrum of compound **8** (TFA salt, D_2O, 400 MHz).
Figure S27. HSQC spectrum of compound **8** (TFA salt, D_2O, 400 MHz).
Figure S28. HMBC spectrum of compound **8** (TFA salt, D_2O, 400 MHz).
Figure S29. NOESY spectrum of compound **8** (600 ms mixing time, TFA salt, D_2O, 400 MHz).

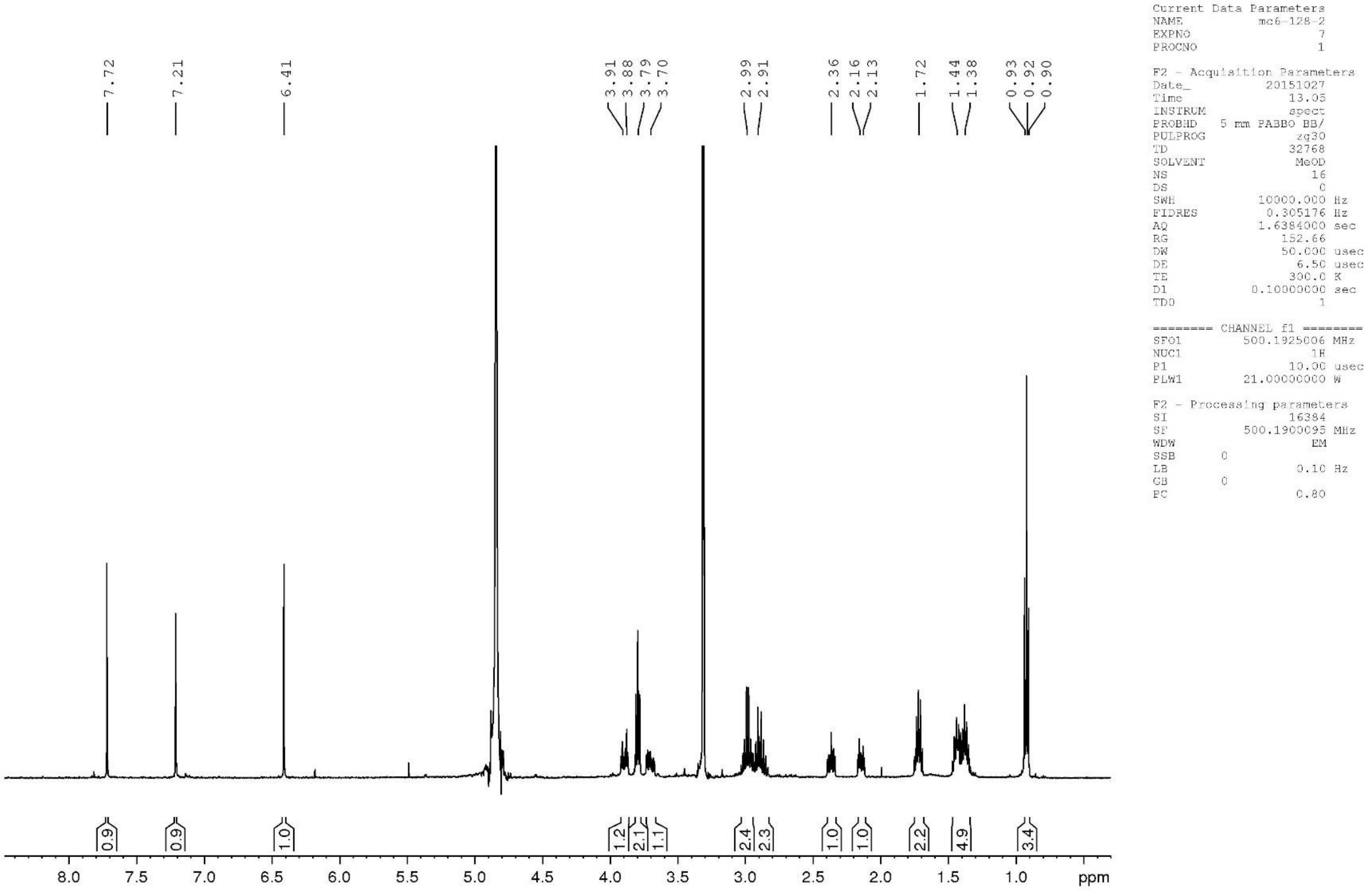

Figure S1. ^{1}H NMR spectrum of compound **4** (TFA salt, CD_3OD, 500 MHz).

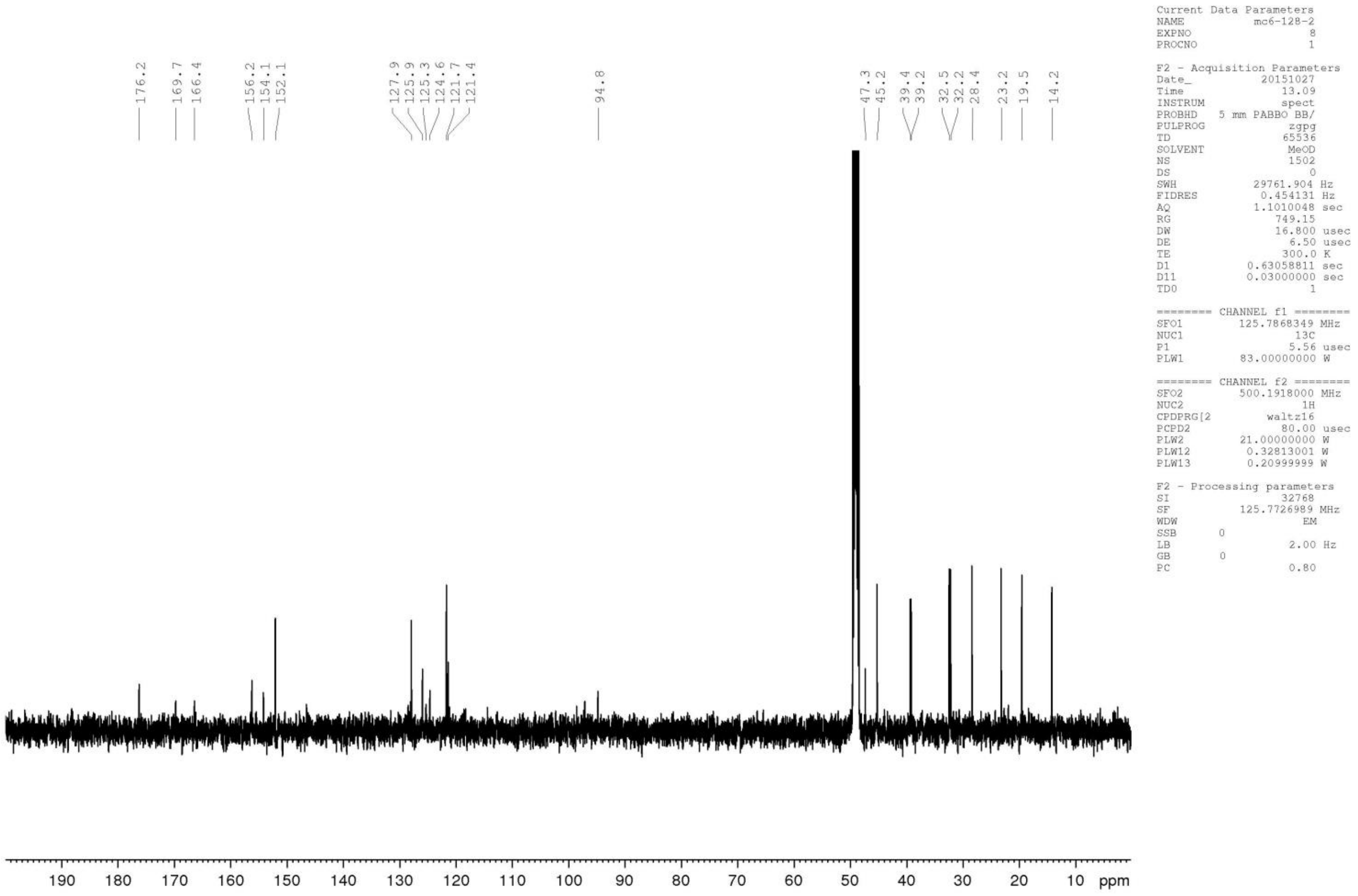

Figure S2. ^{13}C NMR spectrum of compound **4** (TFA salt, CD_3OD, 125 MHz).

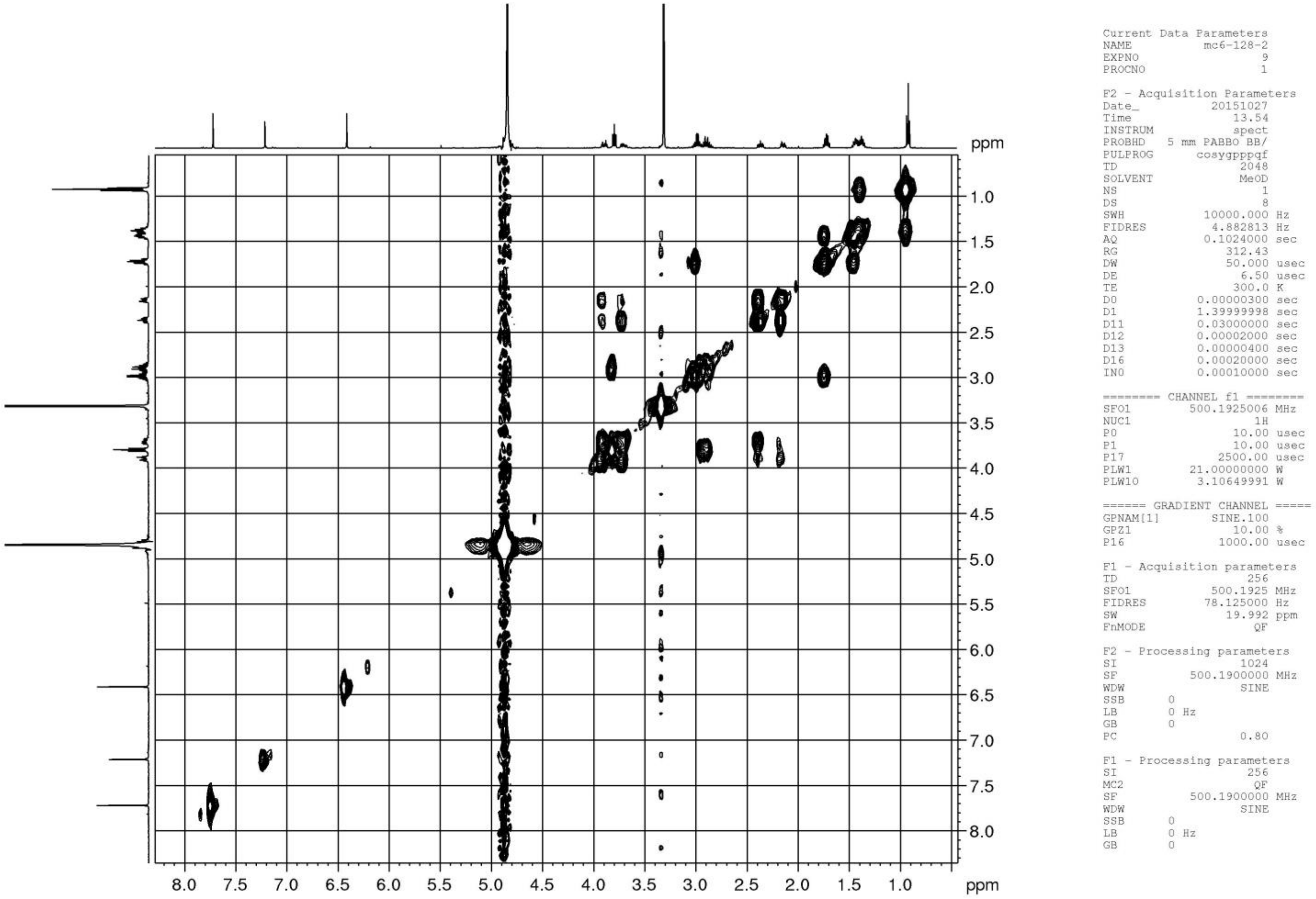

Figure S3. COSY spectrum of compound **4** (TFA salt, CD_3OD, 500 MHz).

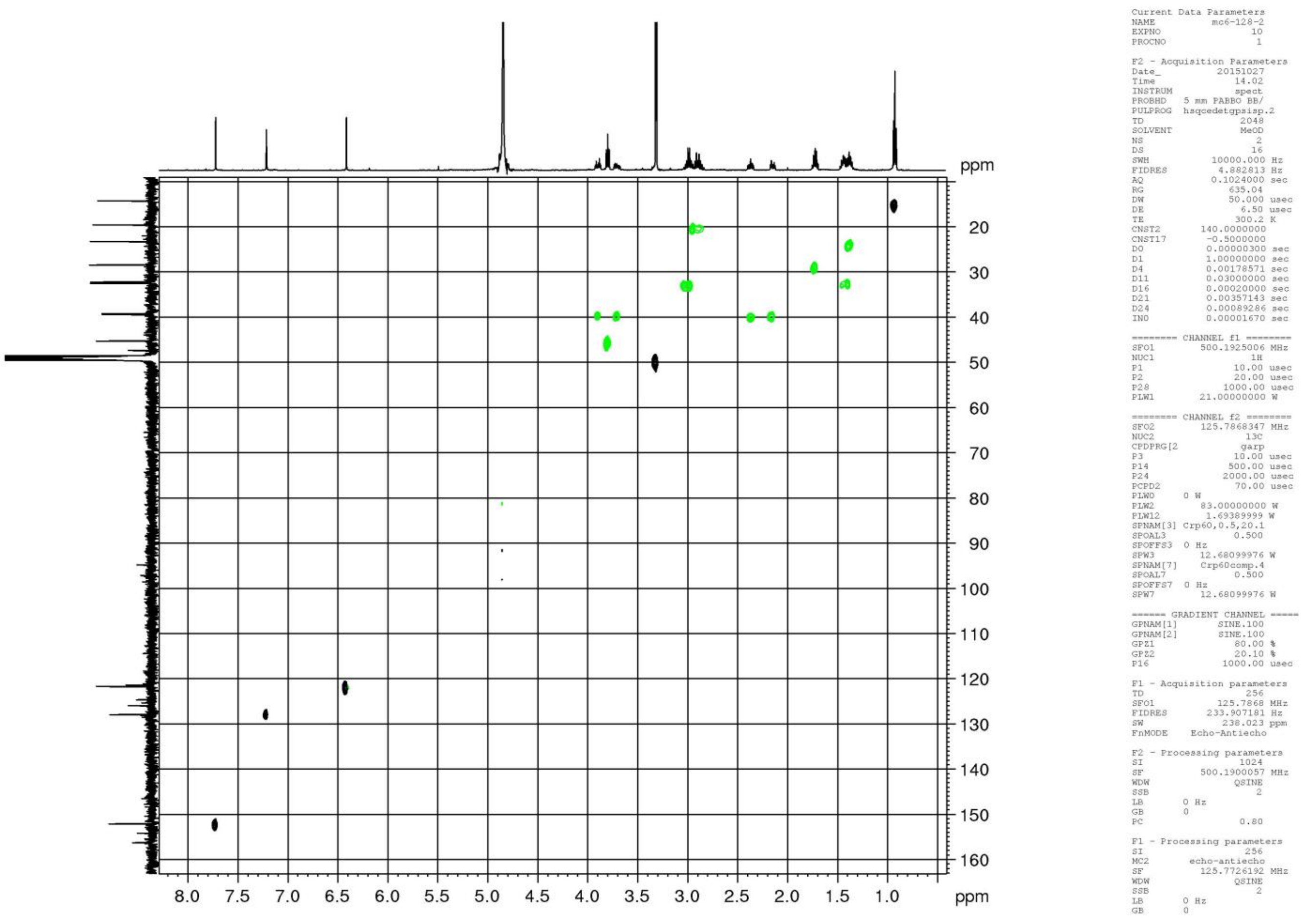

Figure S4. HSQC spectrum of compound **4** (TFA salt, CD_3OD, 500 MHz).

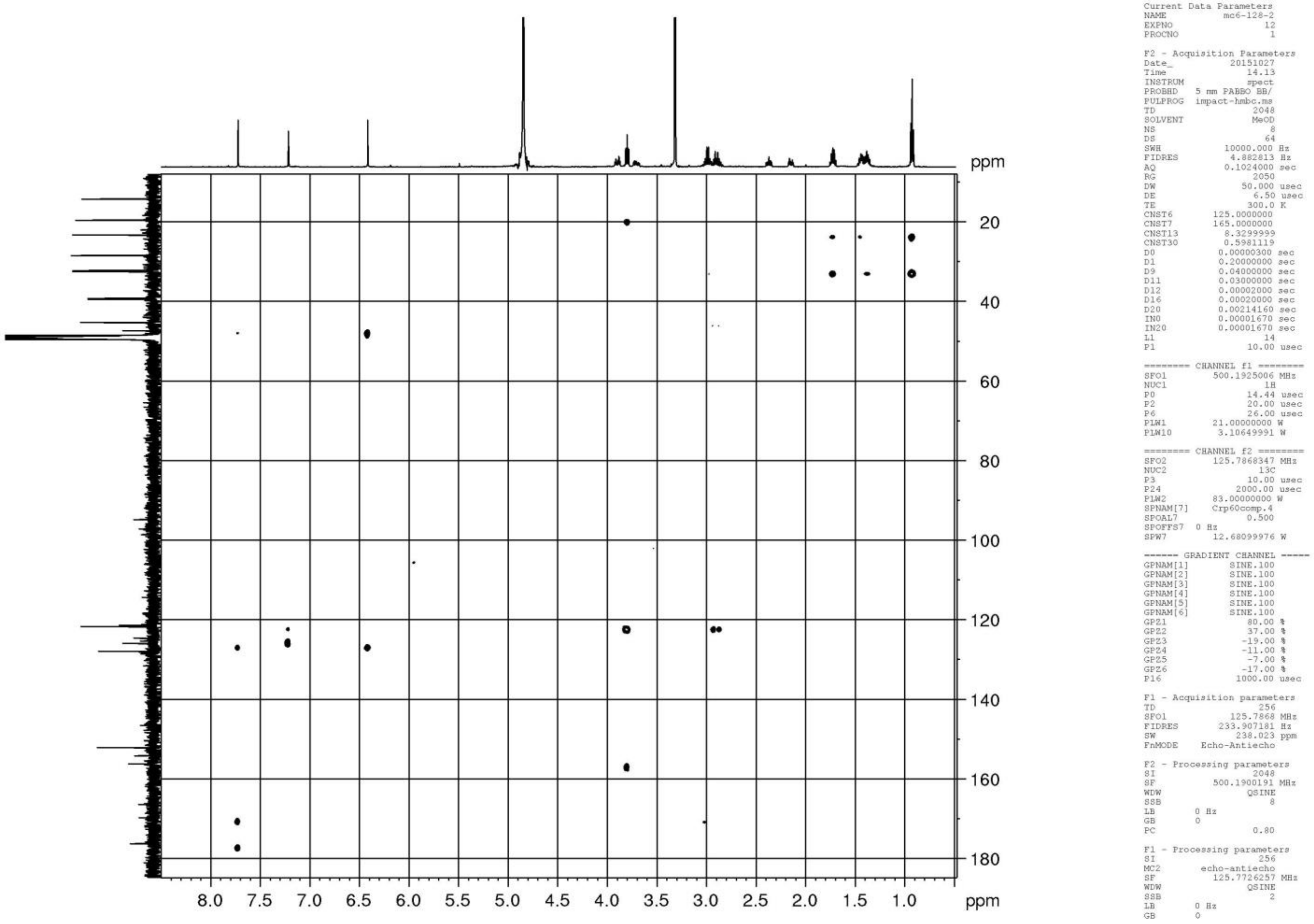

Figure S5. HMBC spectrum of compound **4** (TFA salt, CD_3OD, 500 MHz).

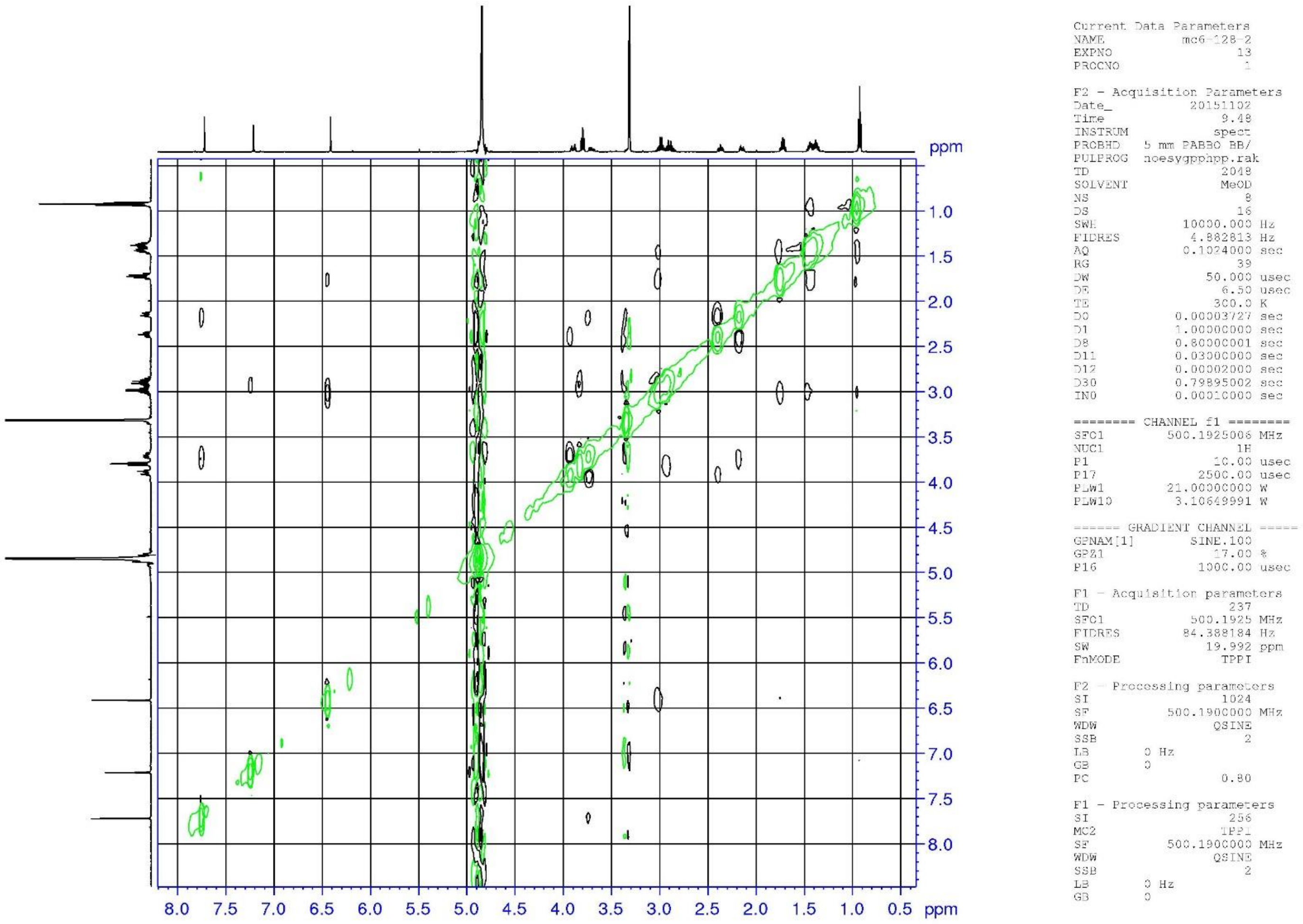

Figure S6. NOESY spectrum of compound **4** (600 ms mixing time, TFA salt, CD_3OD, 500 MHz).

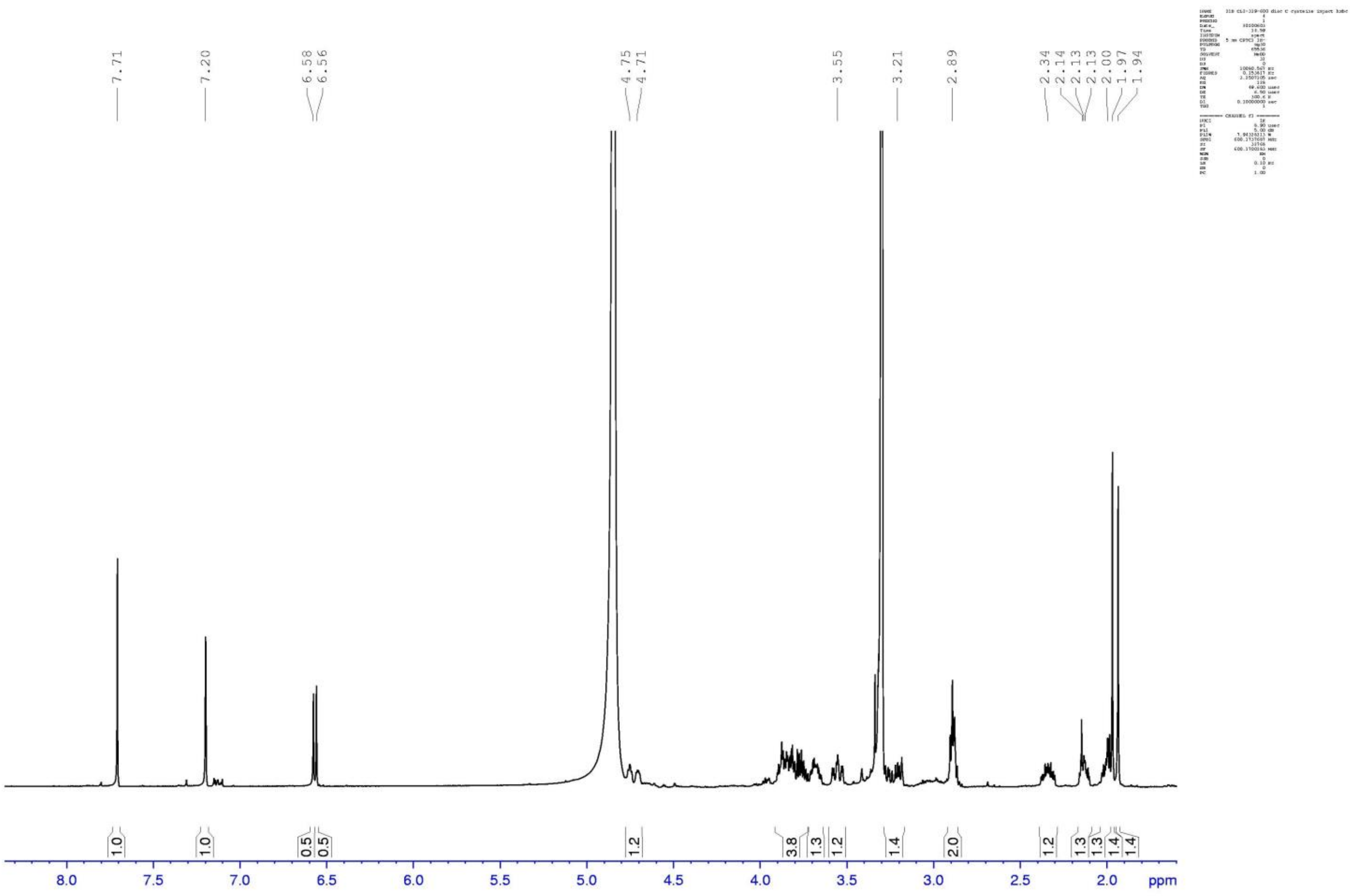

Figure S7. ^{1}H NMR spectrum of compound **5** (TFA salt, CD_3OD, 600 MHz).

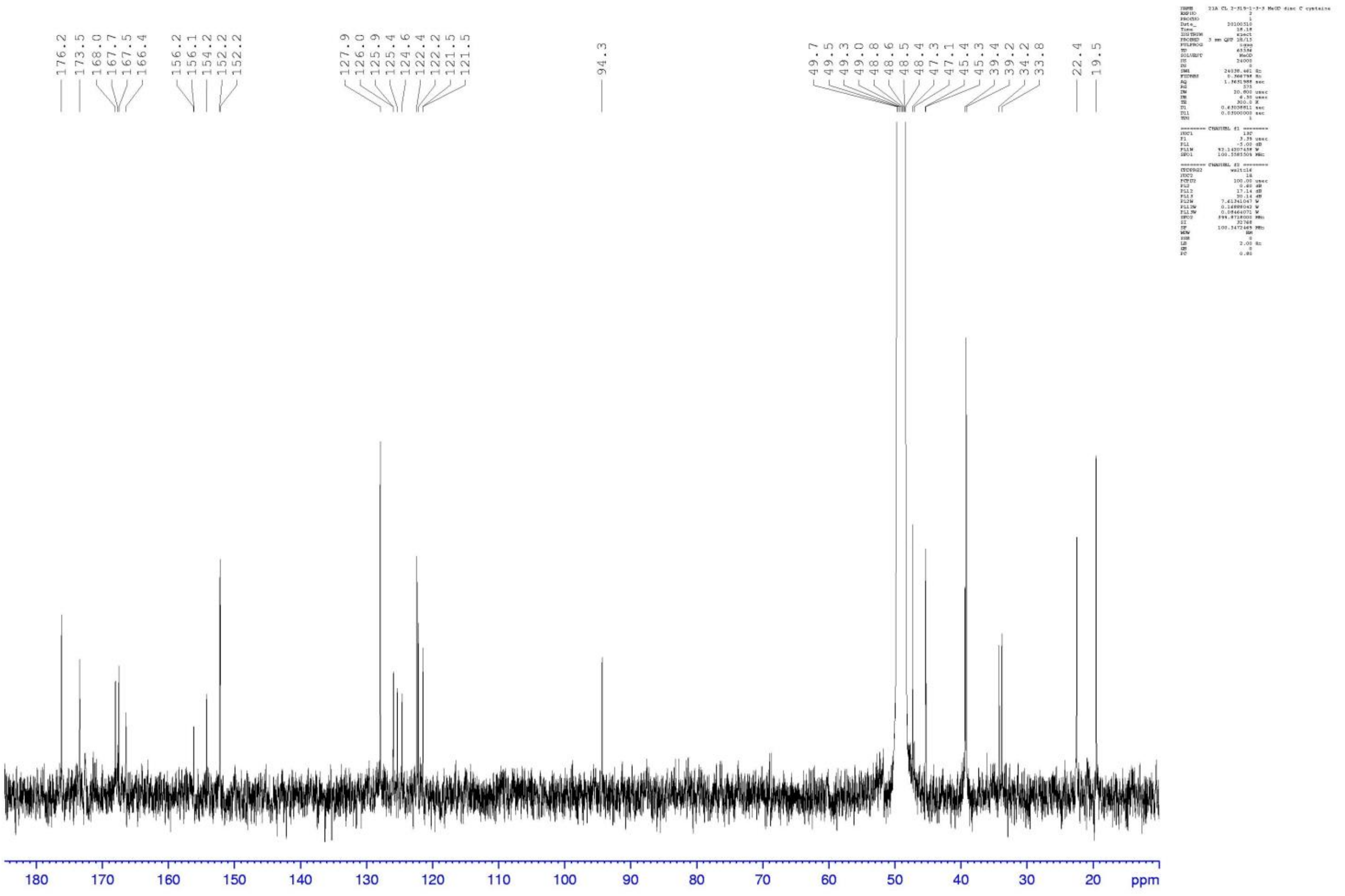

Figure S8. ^{13}C NMR spectrum of compound **5** (TFA salt, CD_3OD, 100 MHz).

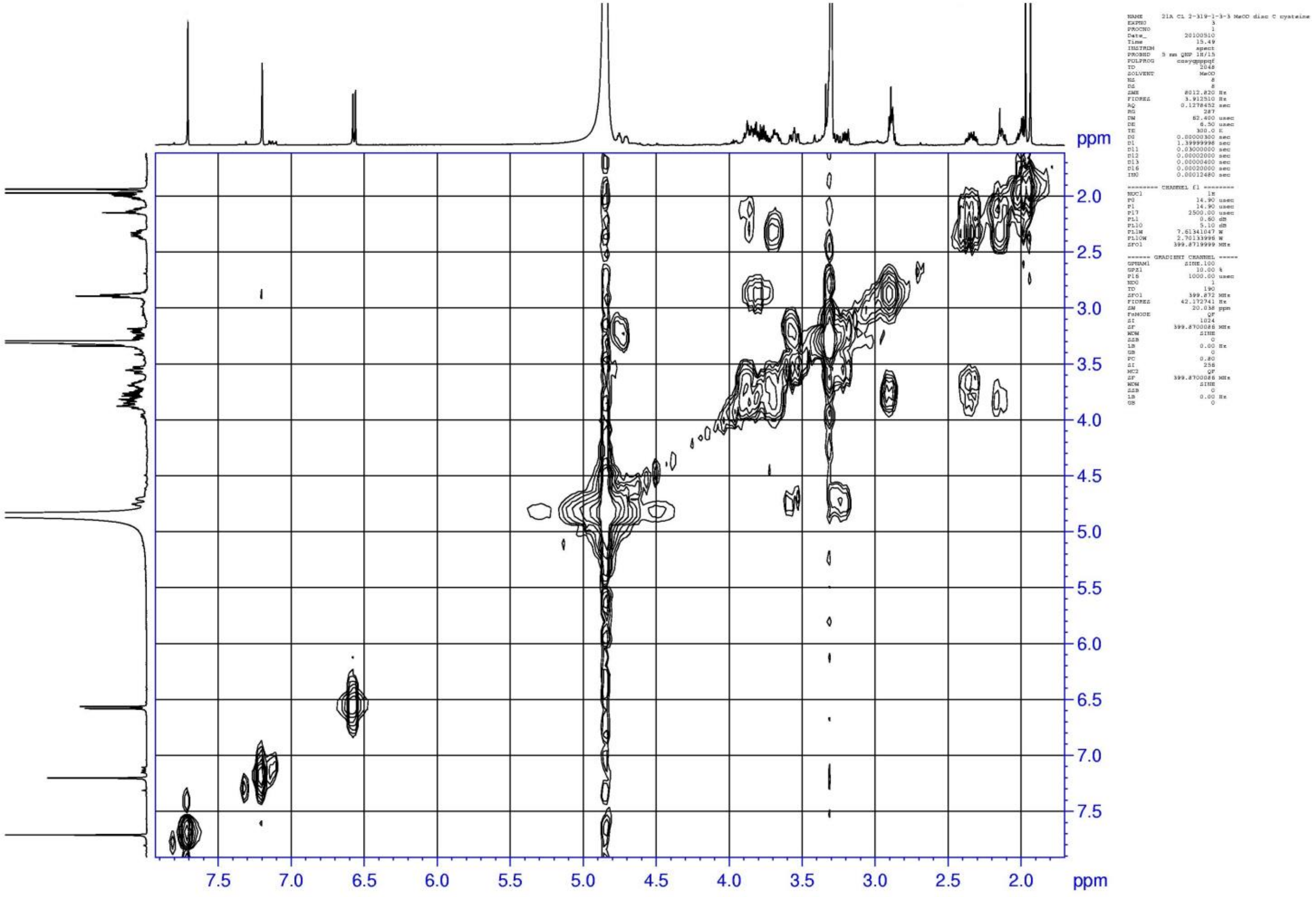

Figure S9. COSY spectrum of compound **5** (TFA salt, CD_3OD, 400 MHz).

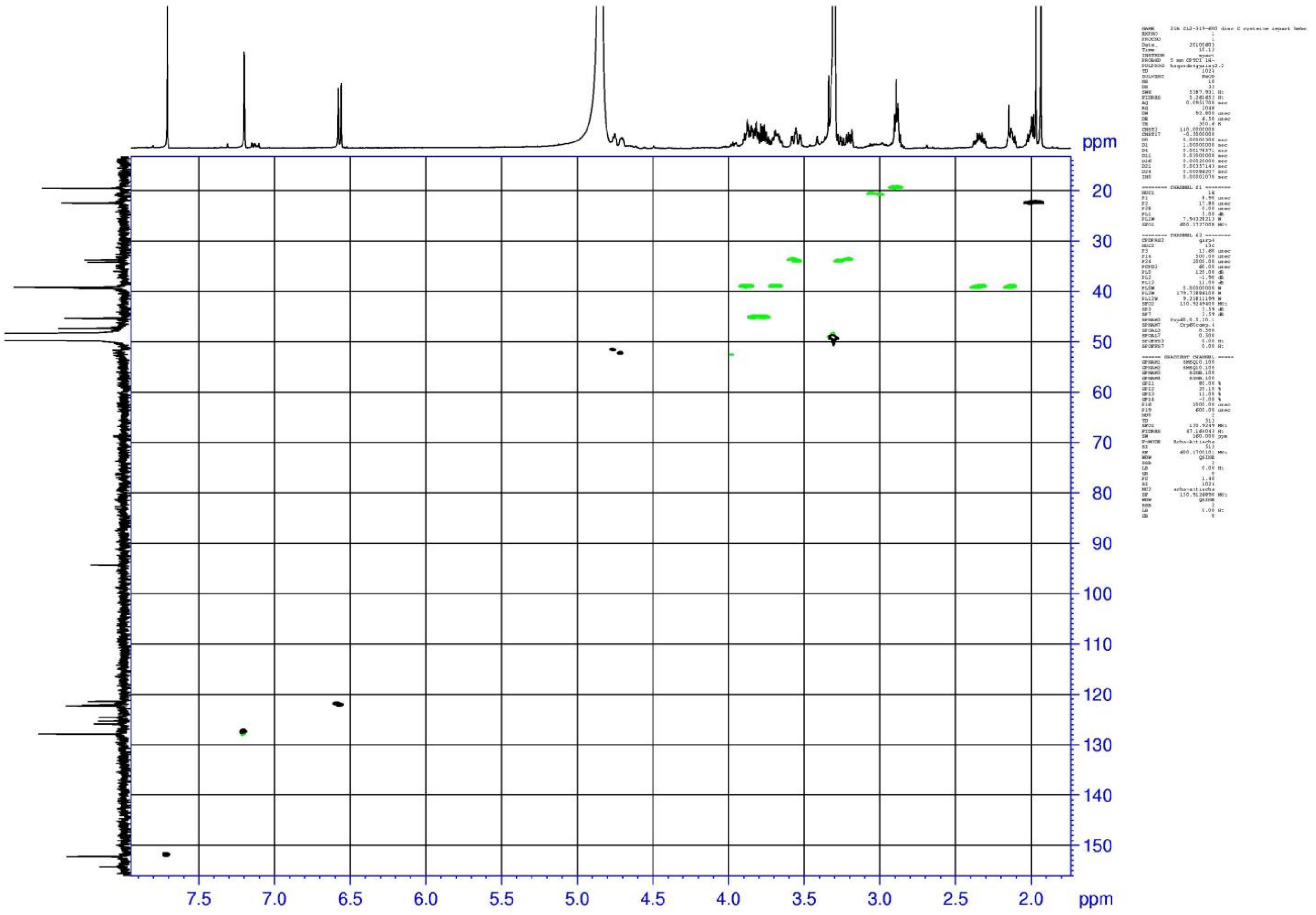

Figure S10. HSQC spectrum of compound **5** (TFA salt, CD_3OD, 600 MHz).

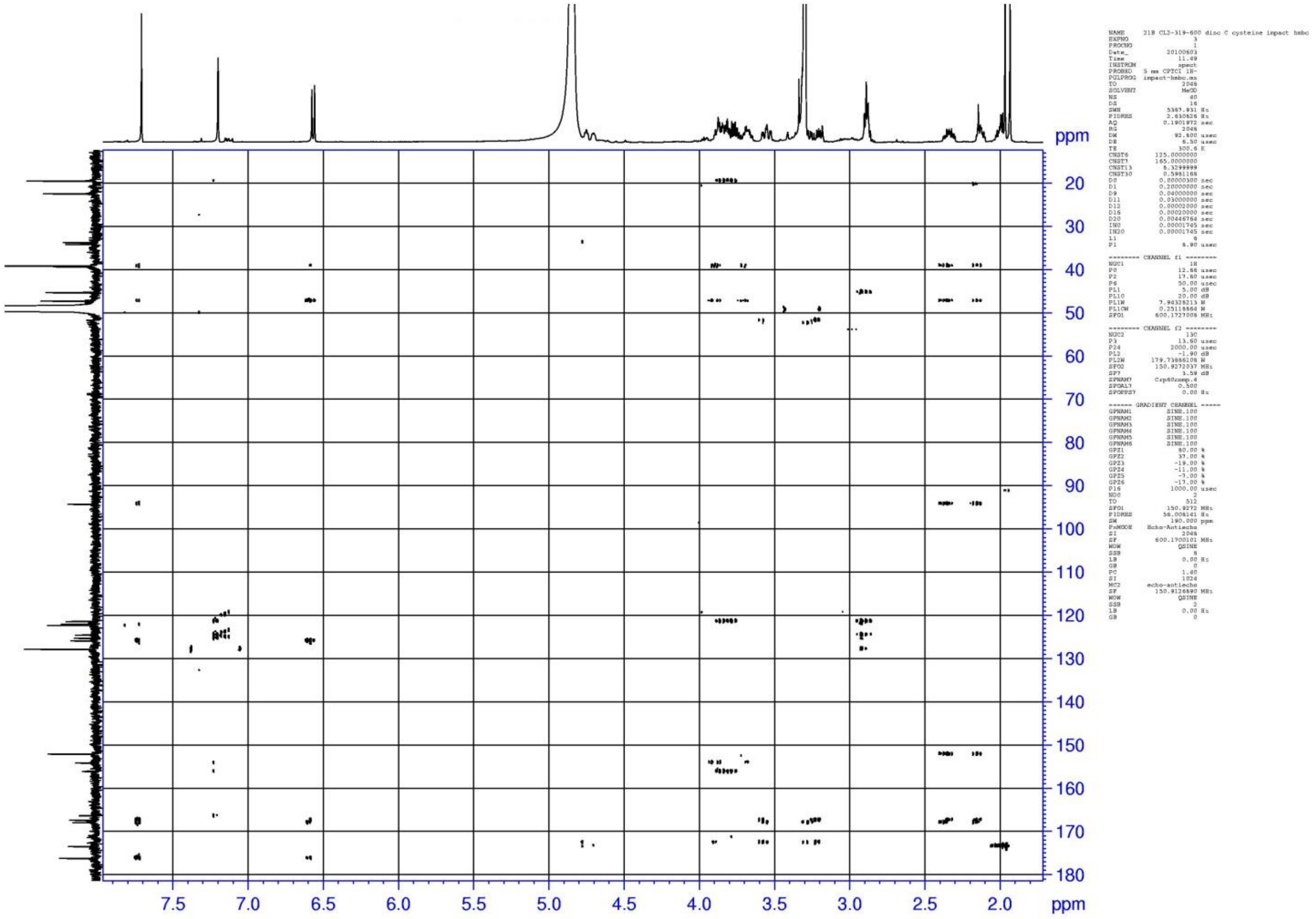

Figure S11. HMBC spectrum of compound **5** (TFA salt, CD_3OD, 600 MHz).

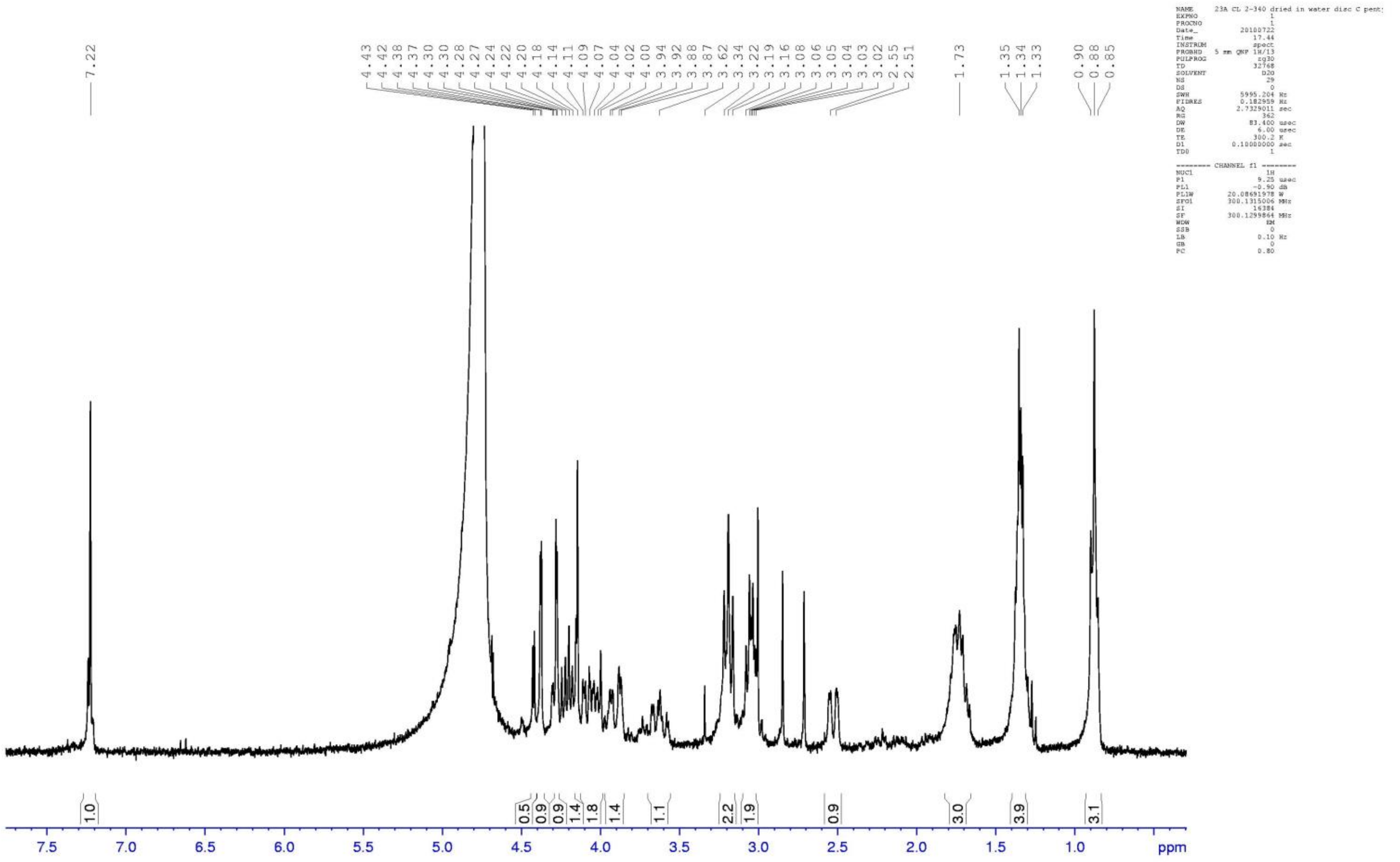

Figure S12. ^{1}H NMR spectrum of compound **6** (TFA salt, D_2O, 400 MHz).

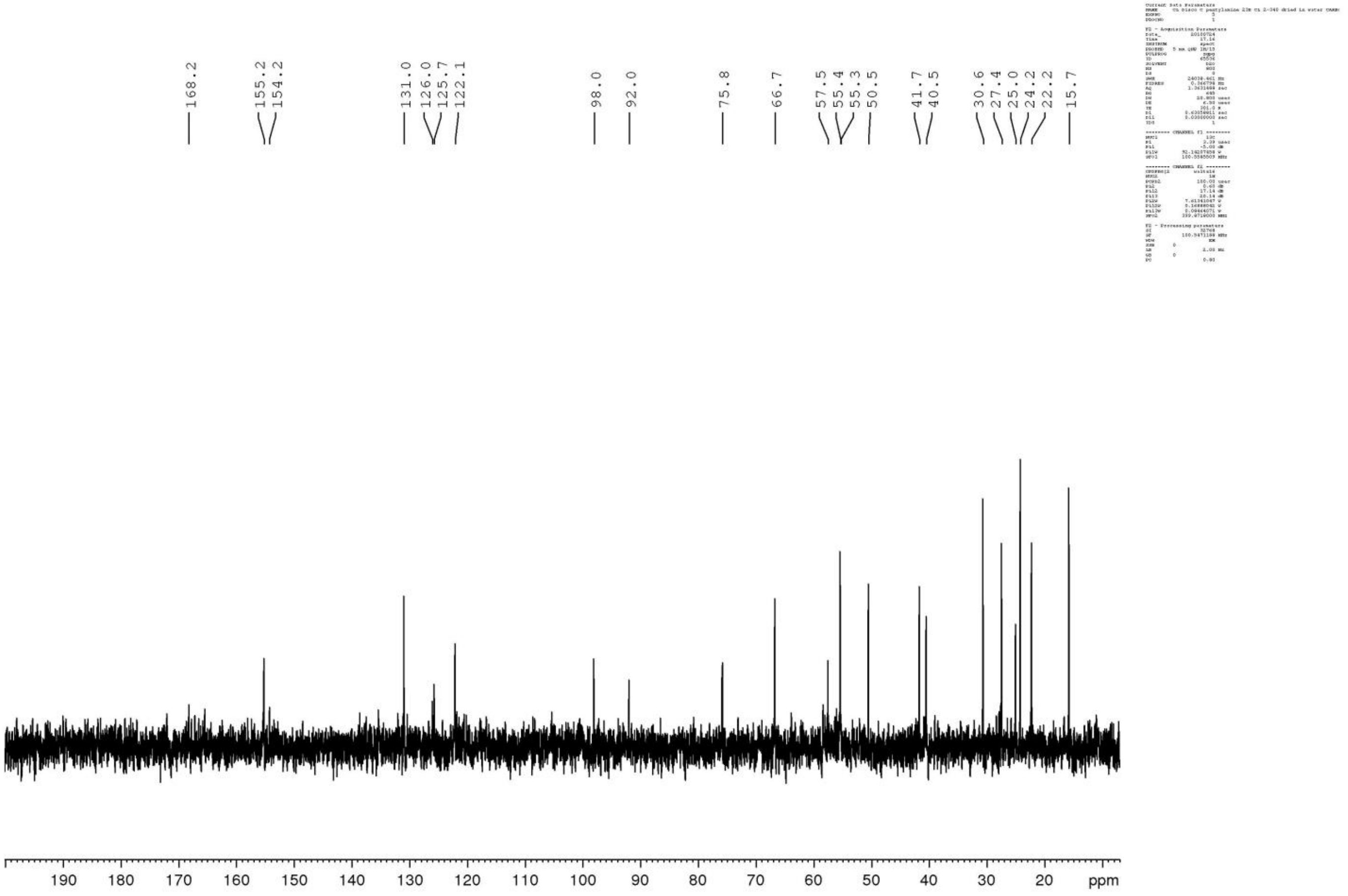

Figure S13. ^{13}C NMR spectrum of compound **6** (TFA salt, D_2O, 100 MHz).

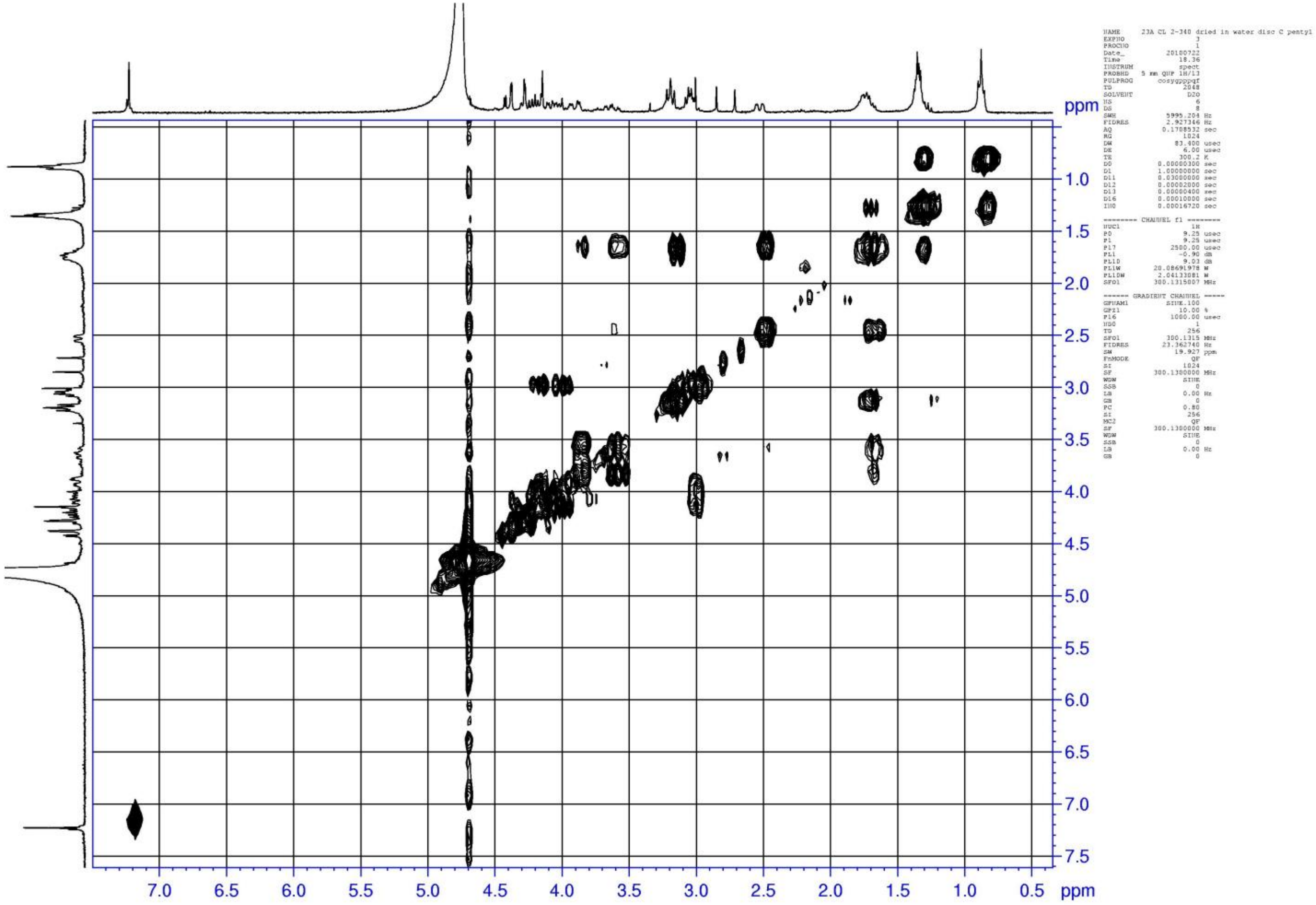

Figure S14. COSY spectrum of compound **6** (TFA salt, D_2O, 400 MHz).

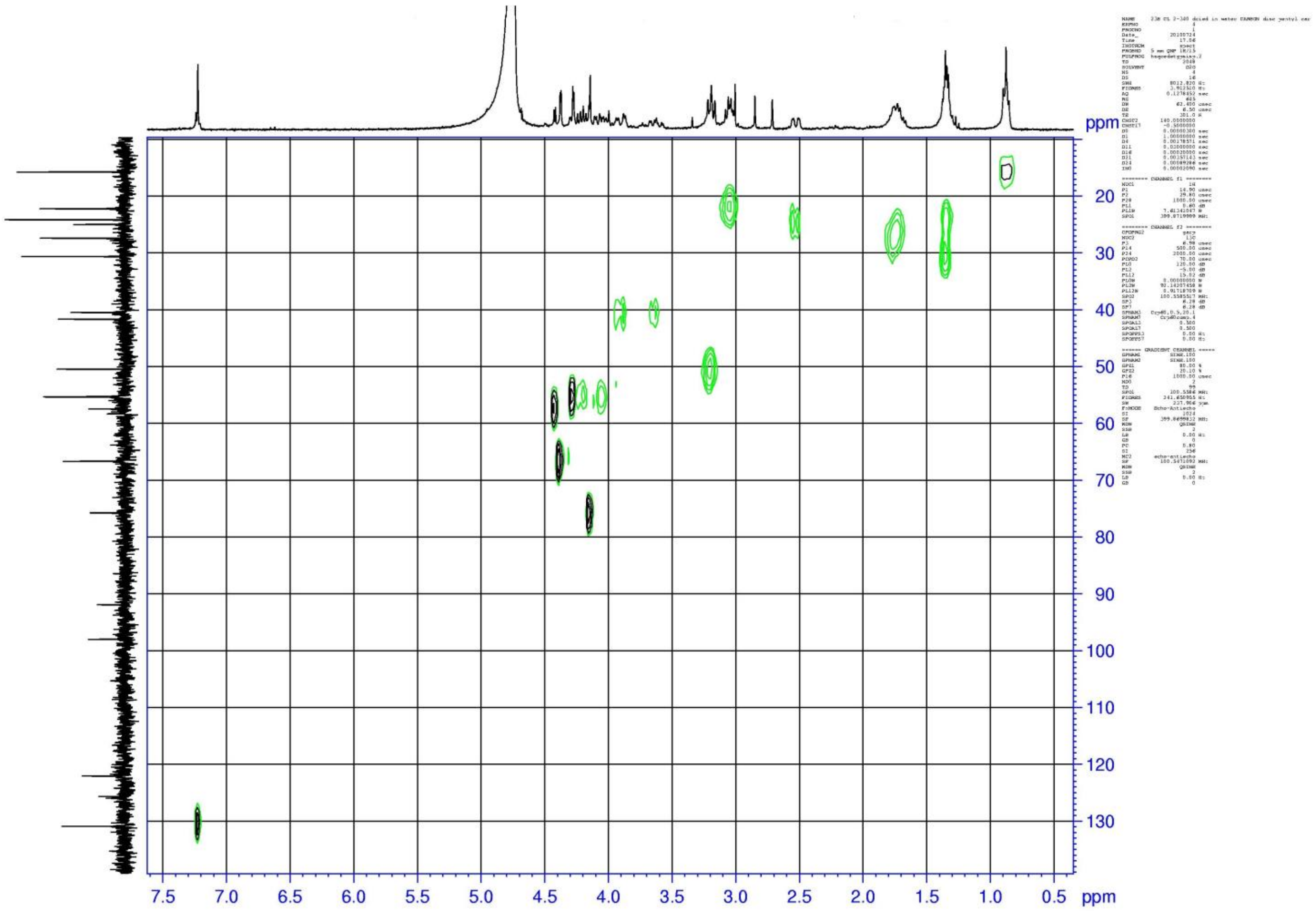

Figure S15. HSQC spectrum of compound **6** (TFA salt, D_2O, 400 MHz).

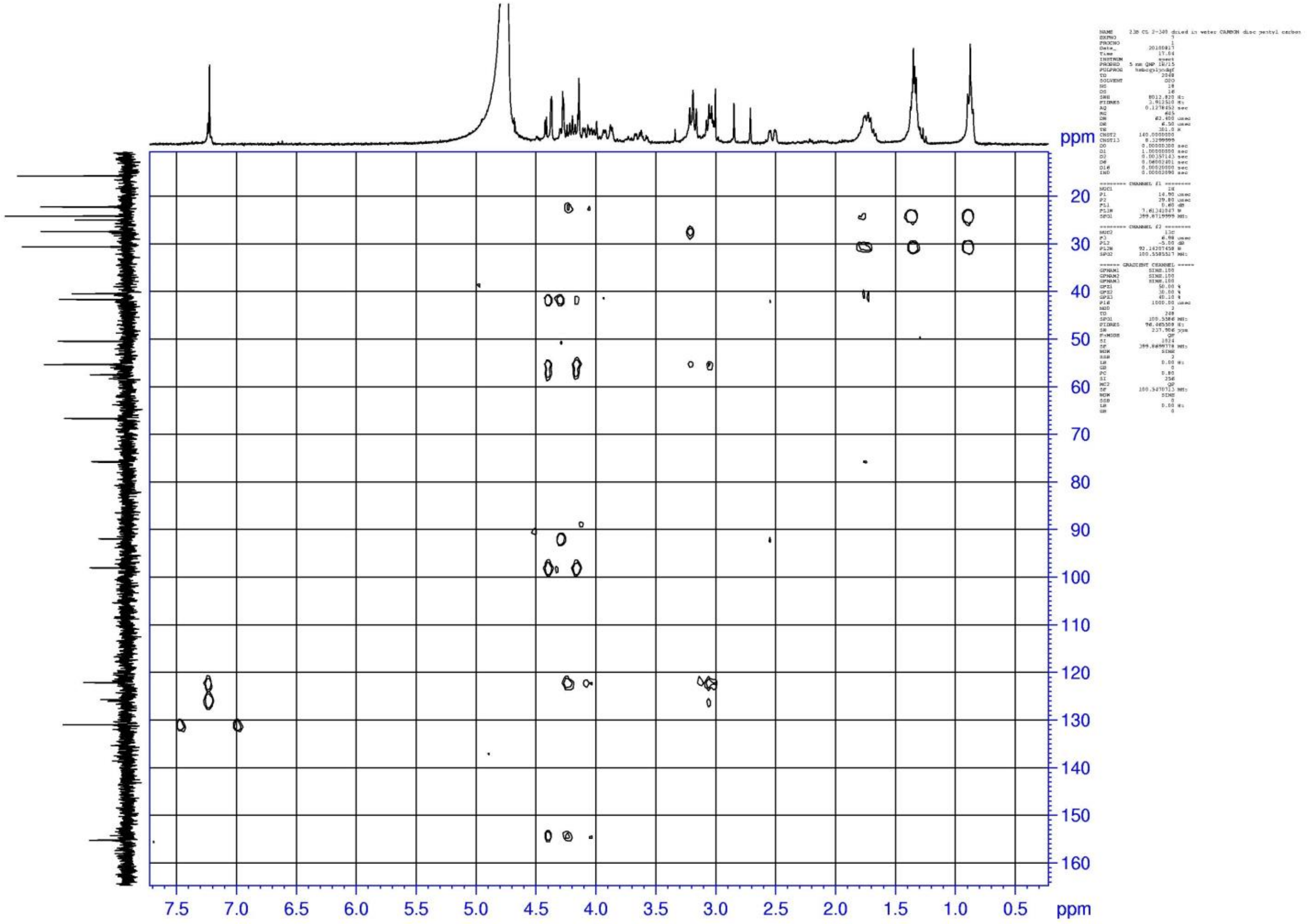

Figure S16. HMBC spectrum of compound **6** (TFA salt, D_2O, 400 MHz).

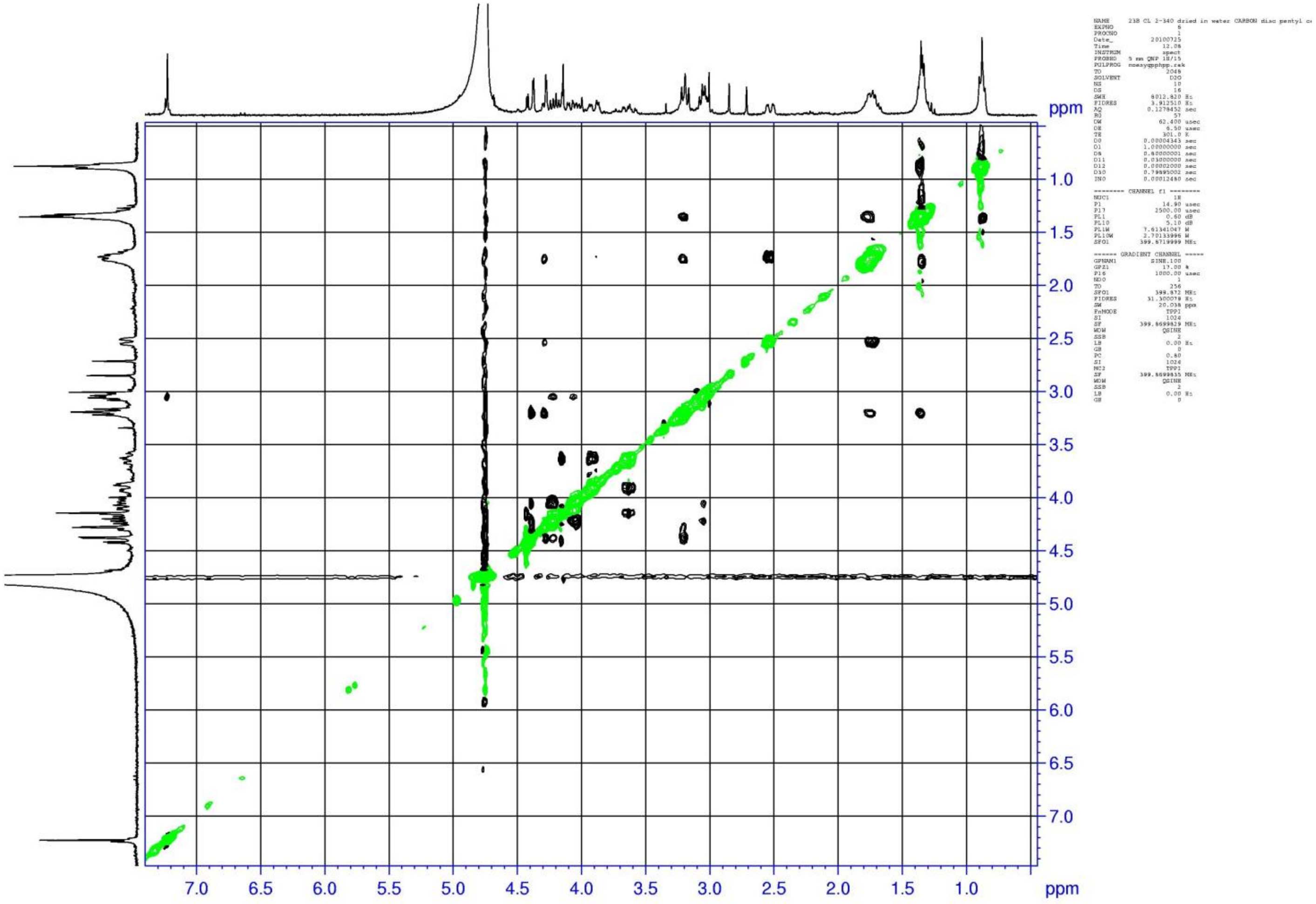

Figure S17. NOESY spectrum of compound **6** (600 ms mixing time, TFA salt, D_2O, 400 MHz).

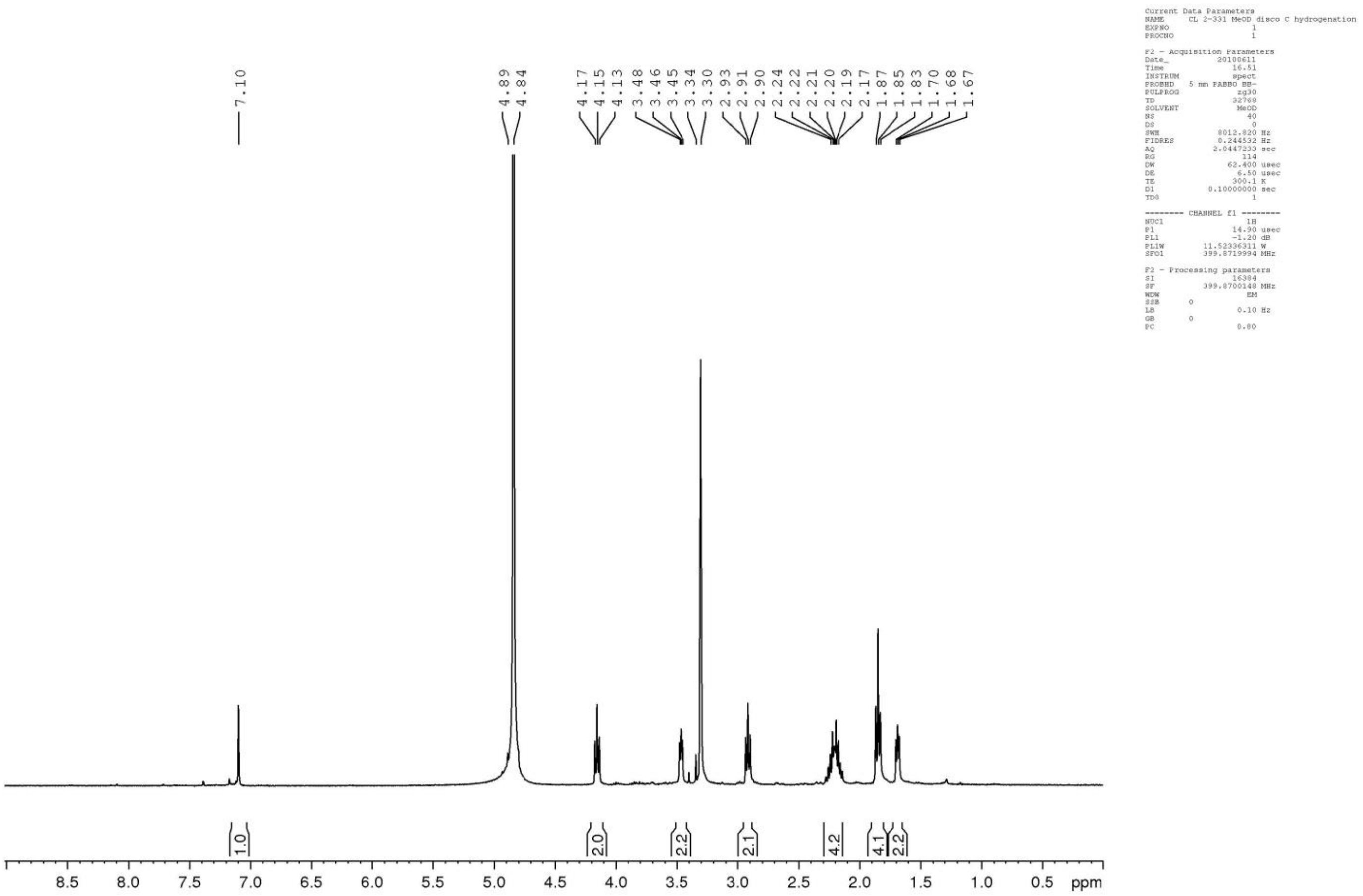

Figure S18. ^{1}H NMR spectrum of compound **7** (TFA salt, CD_3OD, 400 MHz).

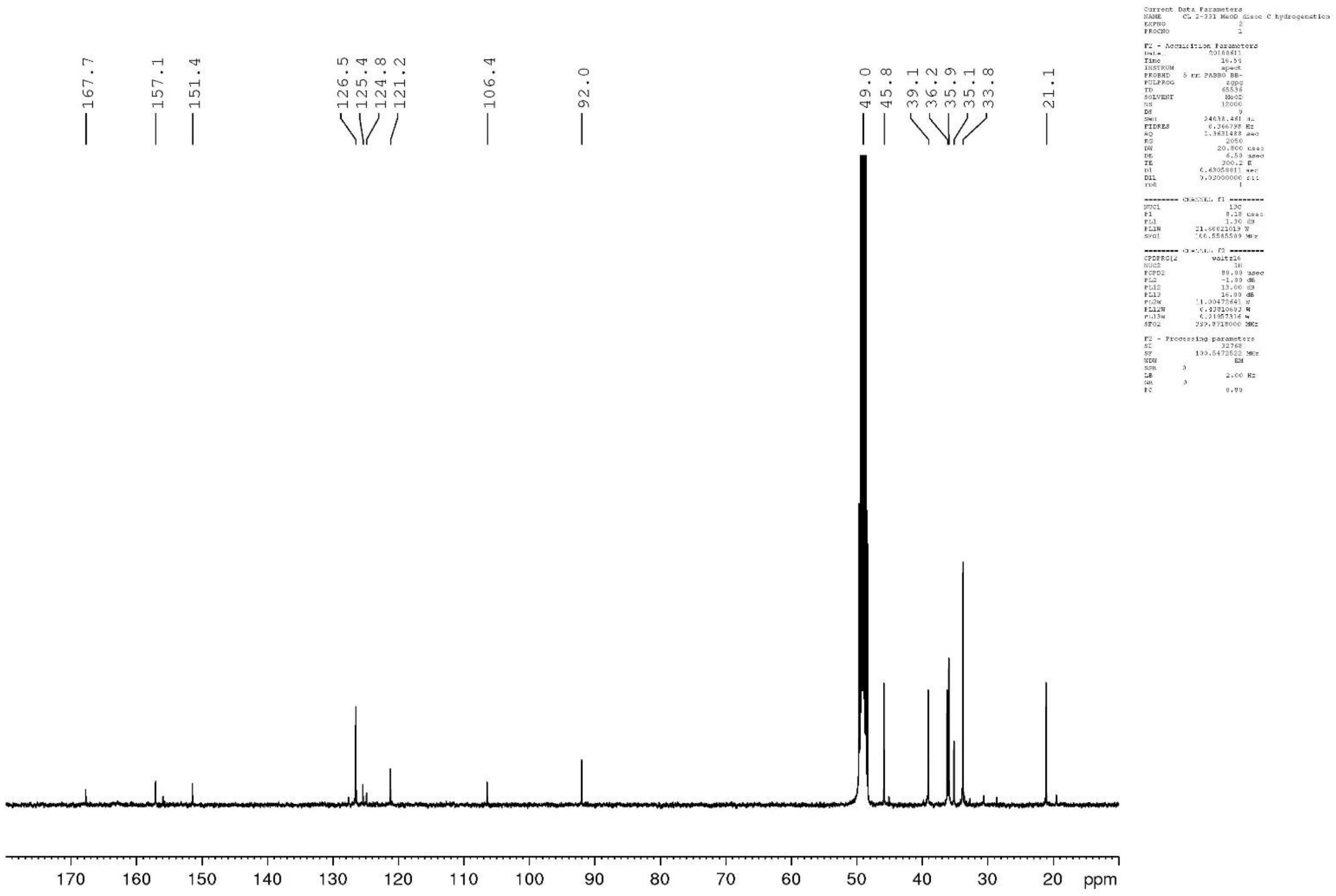

Figure S19. ^{13}C NMR spectrum of compound **7** (TFA salt, CD_3OD, 100 MHz).

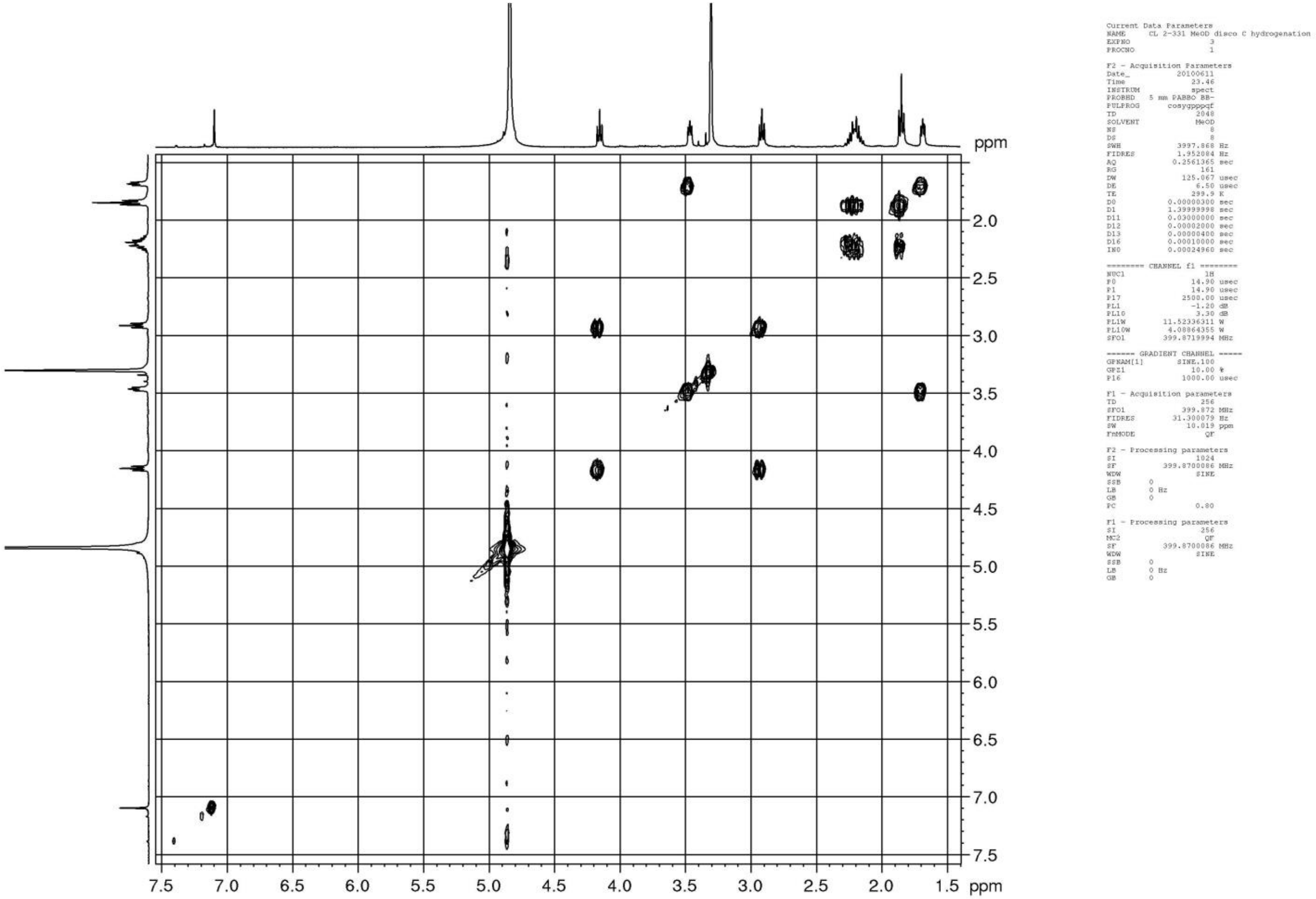

Figure S20. COSY spectrum of compound **7** (TFA salt, CD_3OD, 400 MHz).

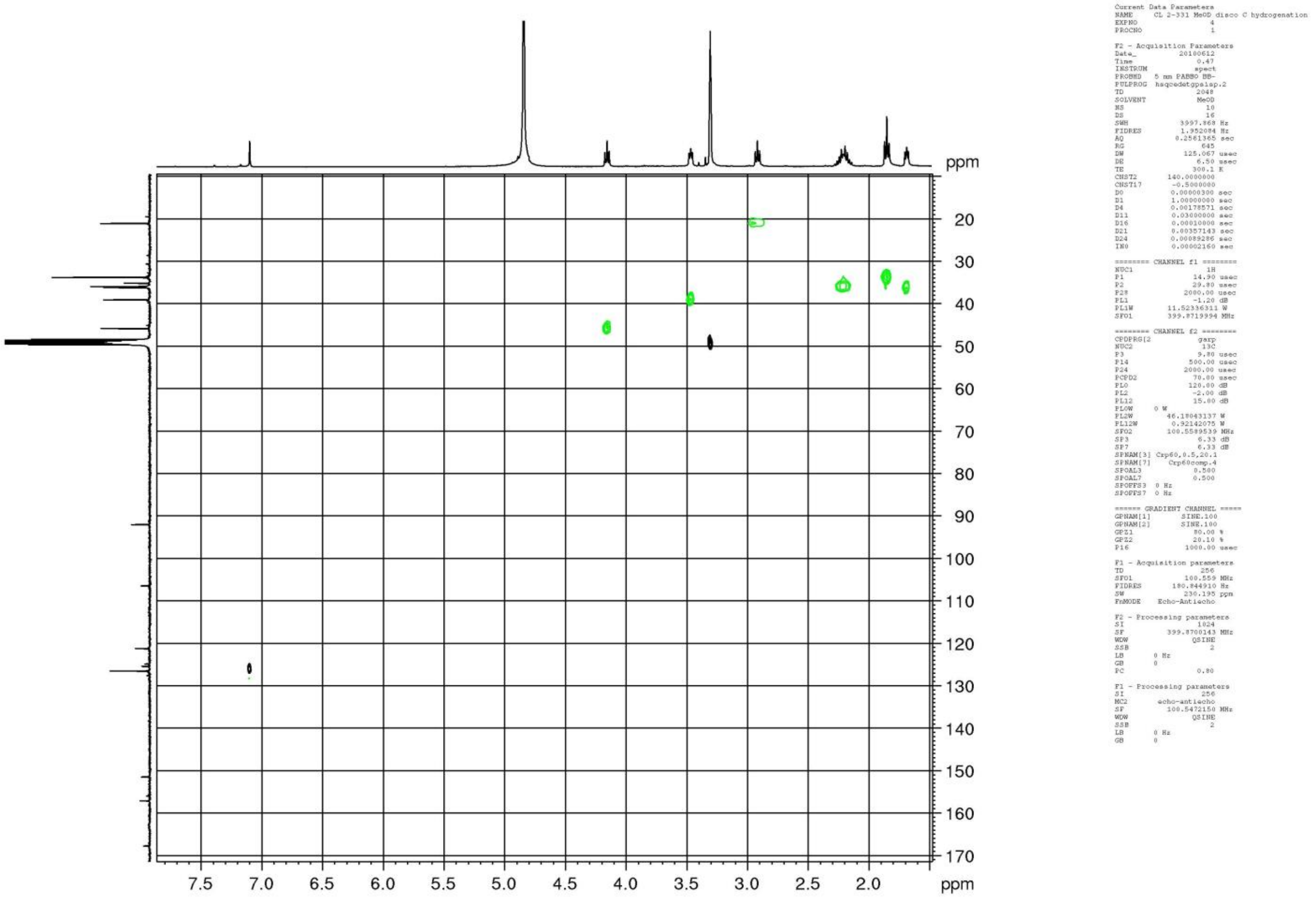

Figure S21. HSQC spectrum of compound **7** (TFA salt, CD_3OD, 400 MHz).

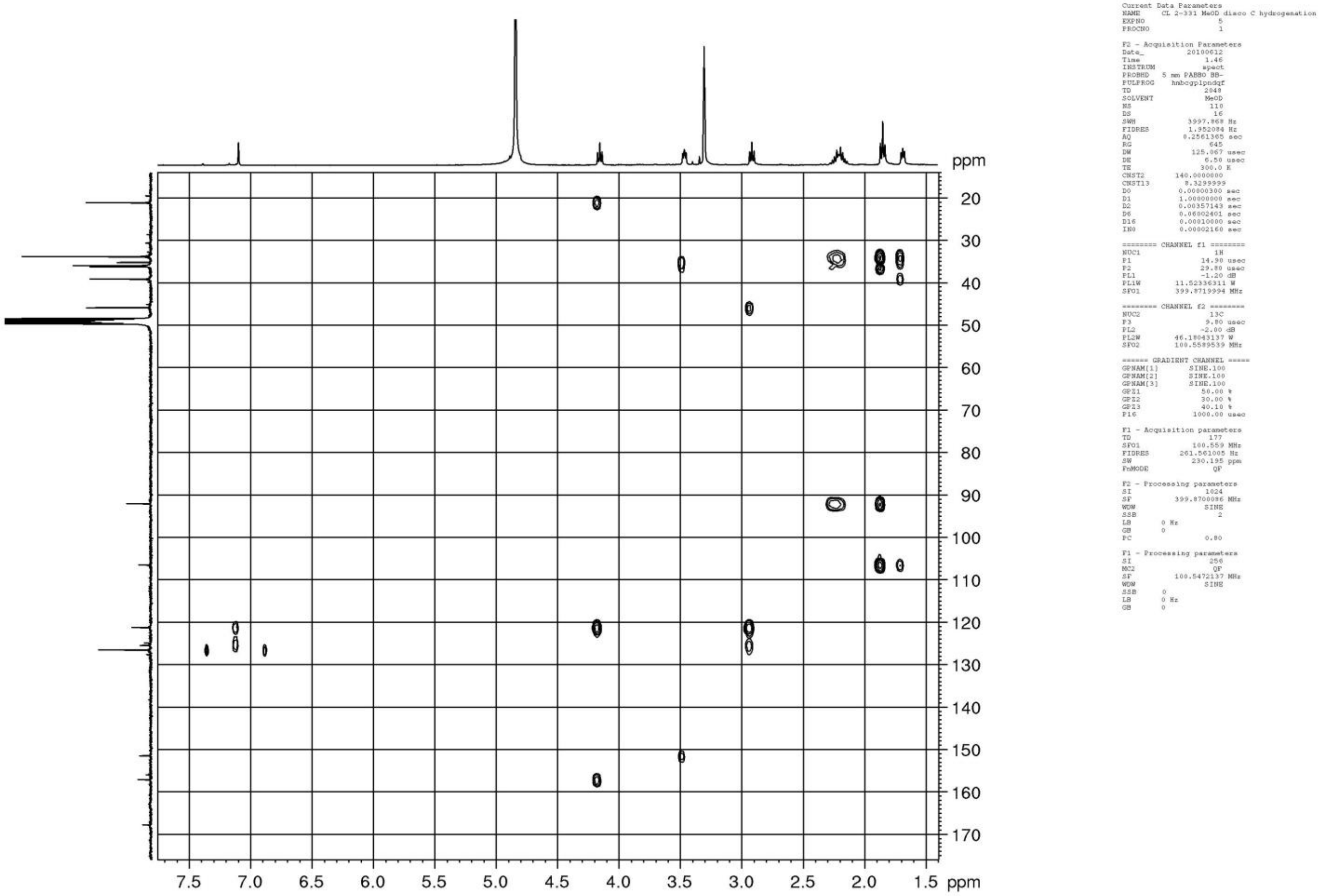

Figure S22. HMBC spectrum of compound **7** (TFA salt, CD_3OD, 400 MHz).

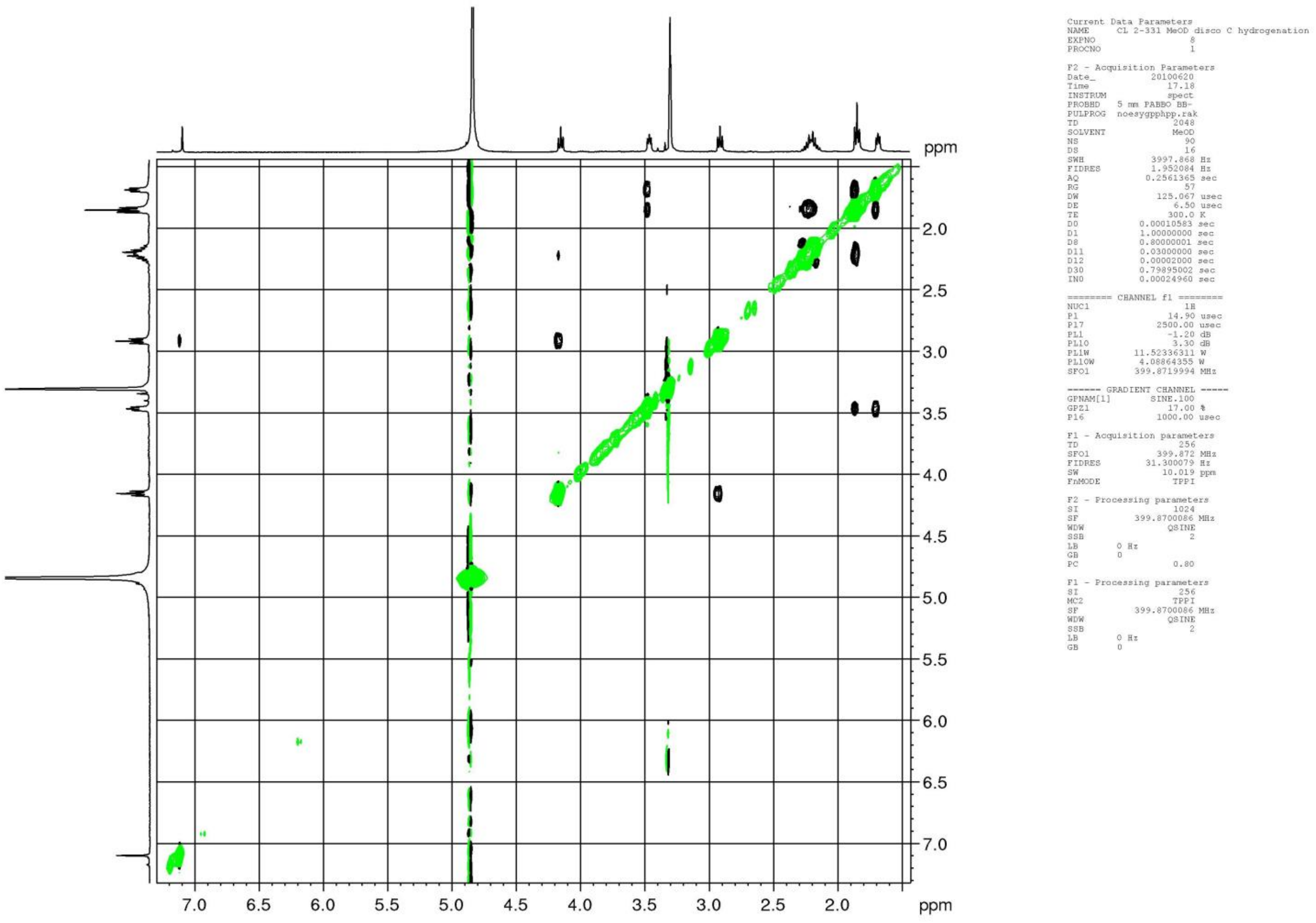

Figure S23. NOESY spectrum of compound **7** (600 ms mixing time, TFA salt, CD_3OD, 400 MHz).

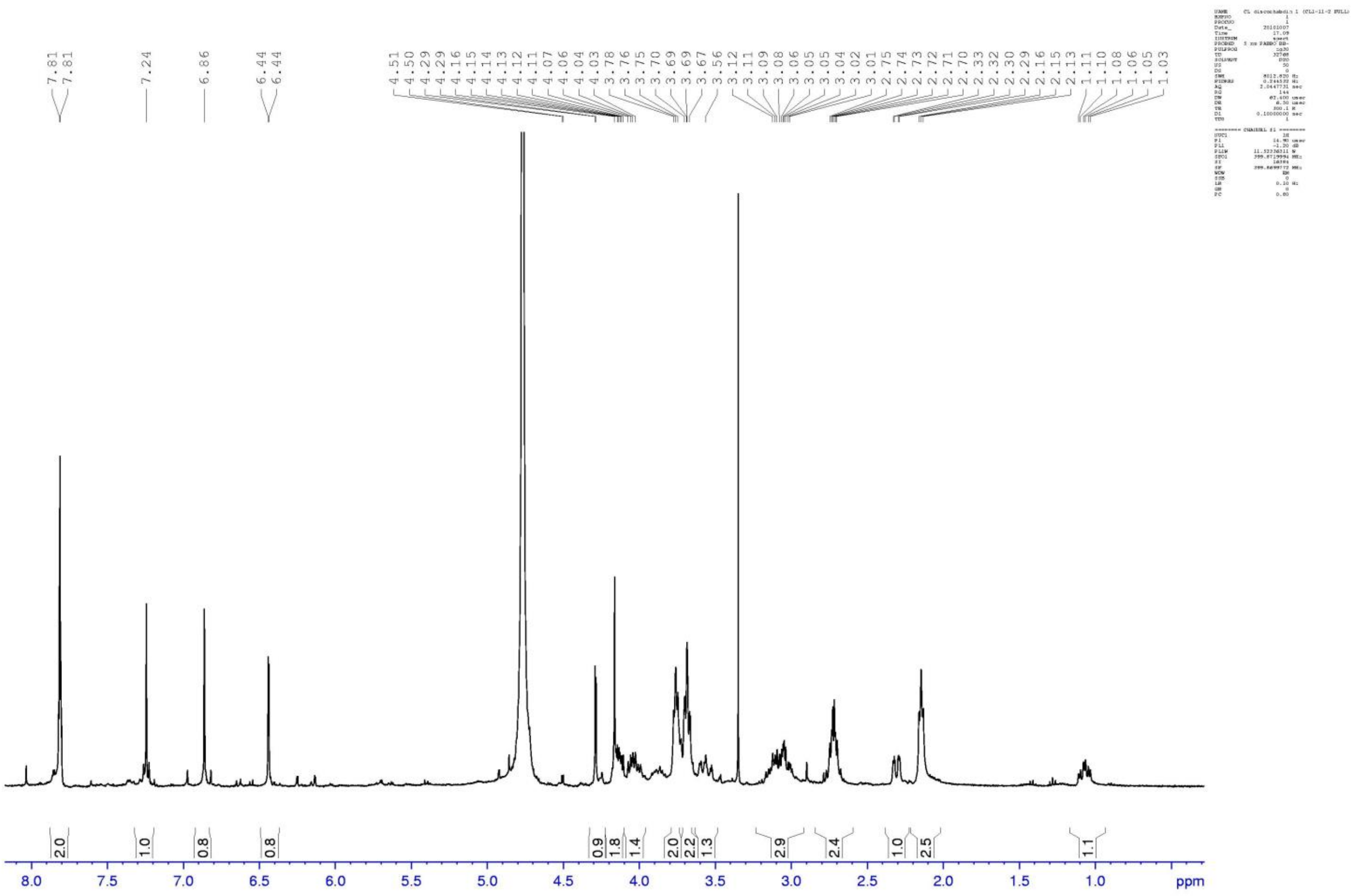

Figure S24. ^{1}H NMR spectrum of compound **8** (TFA salt, D_2O, 400 MHz).

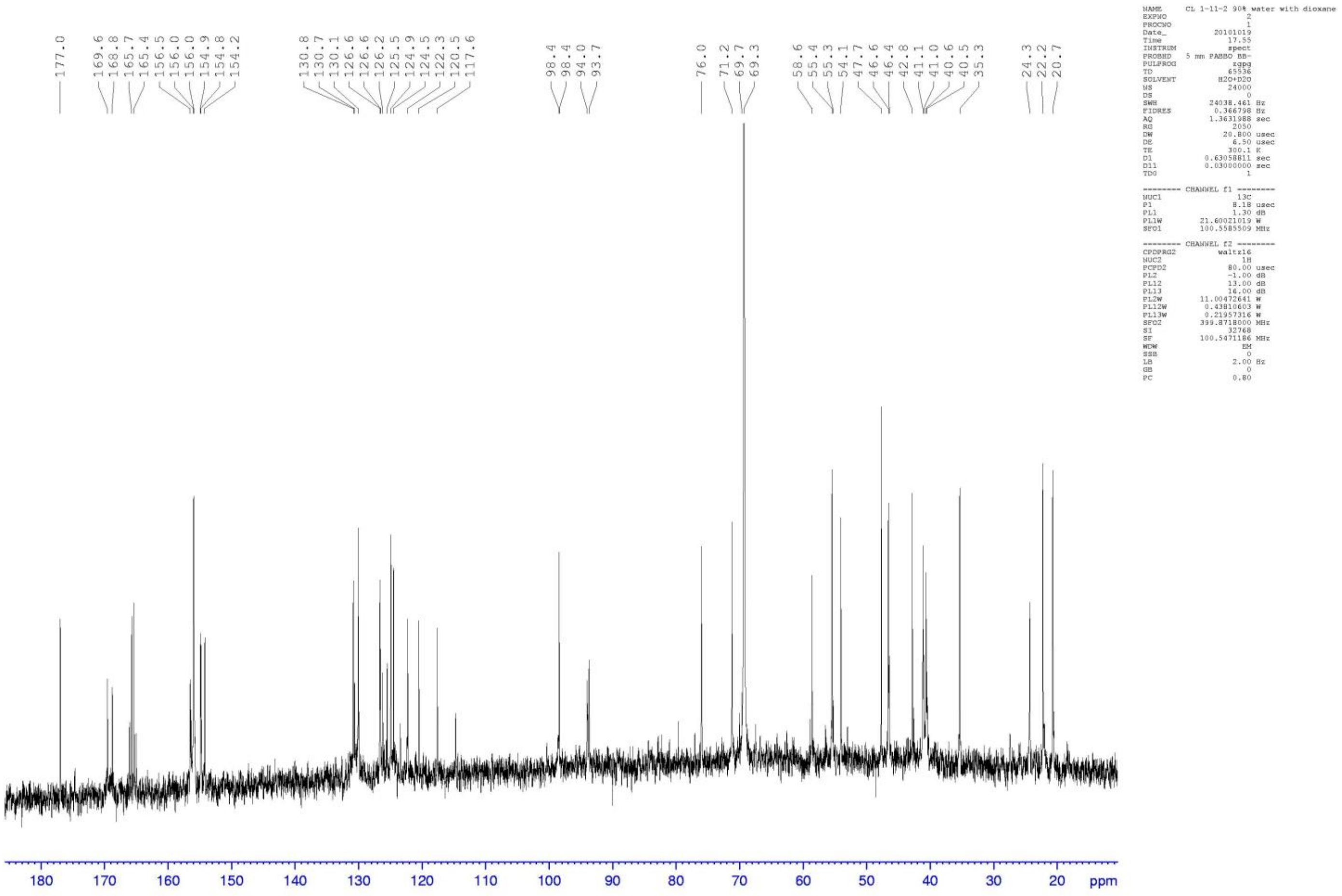

Figure S25. ^{13}C NMR spectrum of compound **8** (TFA salt, 90% H_2O : 10% D_2O, 100 MHz).

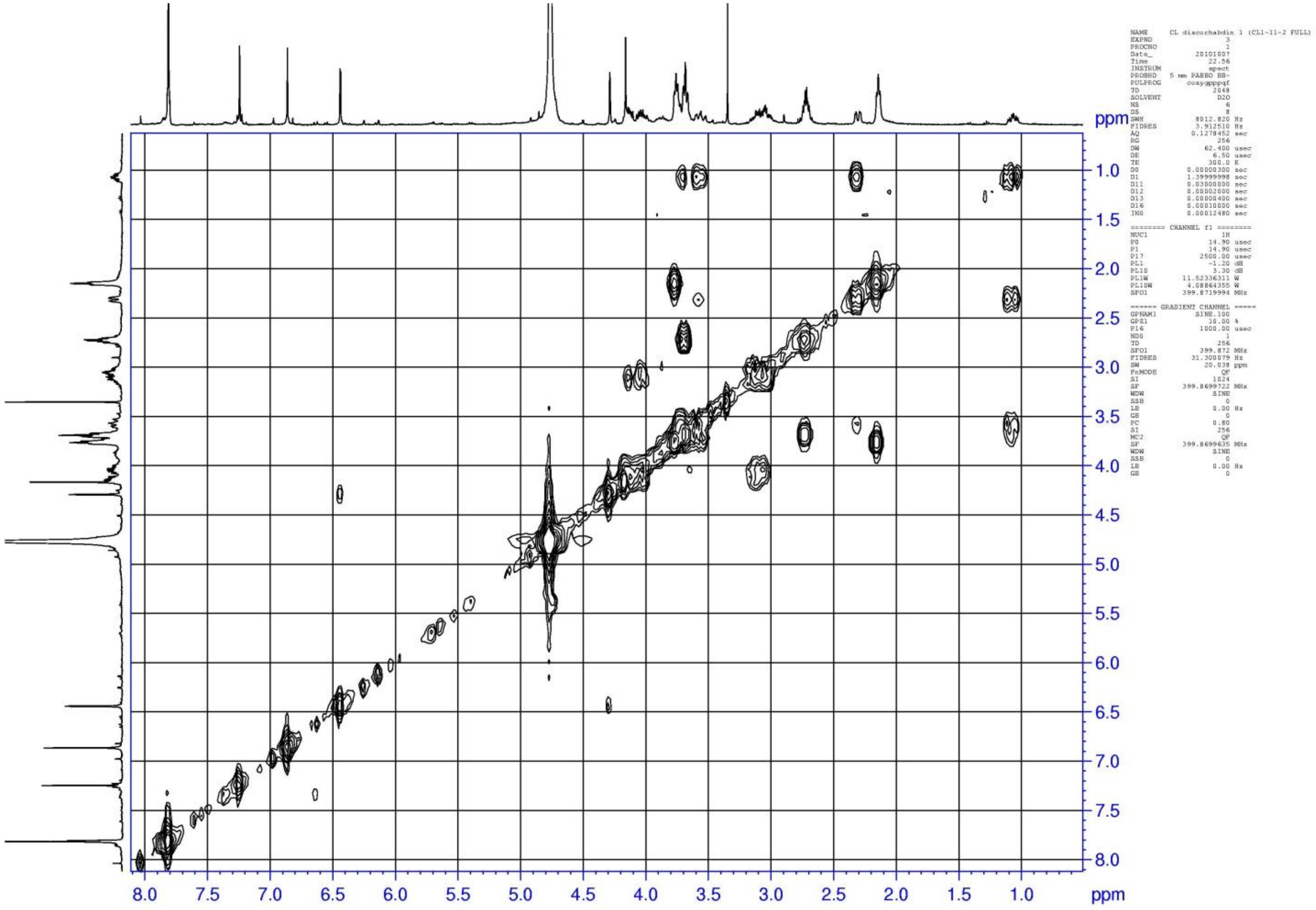

Figure S26. COSY spectrum of compound **8** (TFA salt, D_2O, 400 MHz).

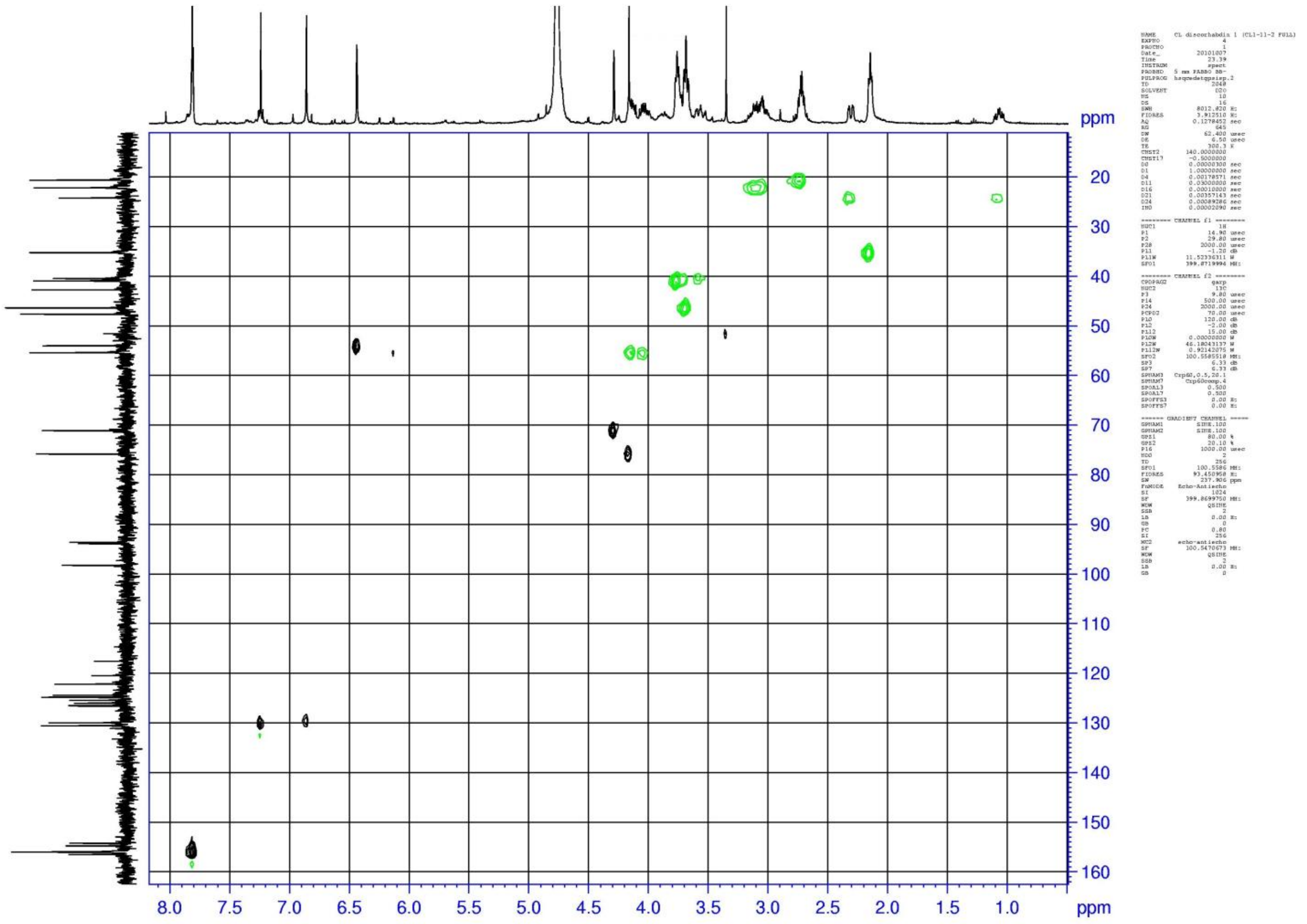

Figure S27. HSQC spectrum of compound **8** (TFA salt, D_2O, 400 MHz).

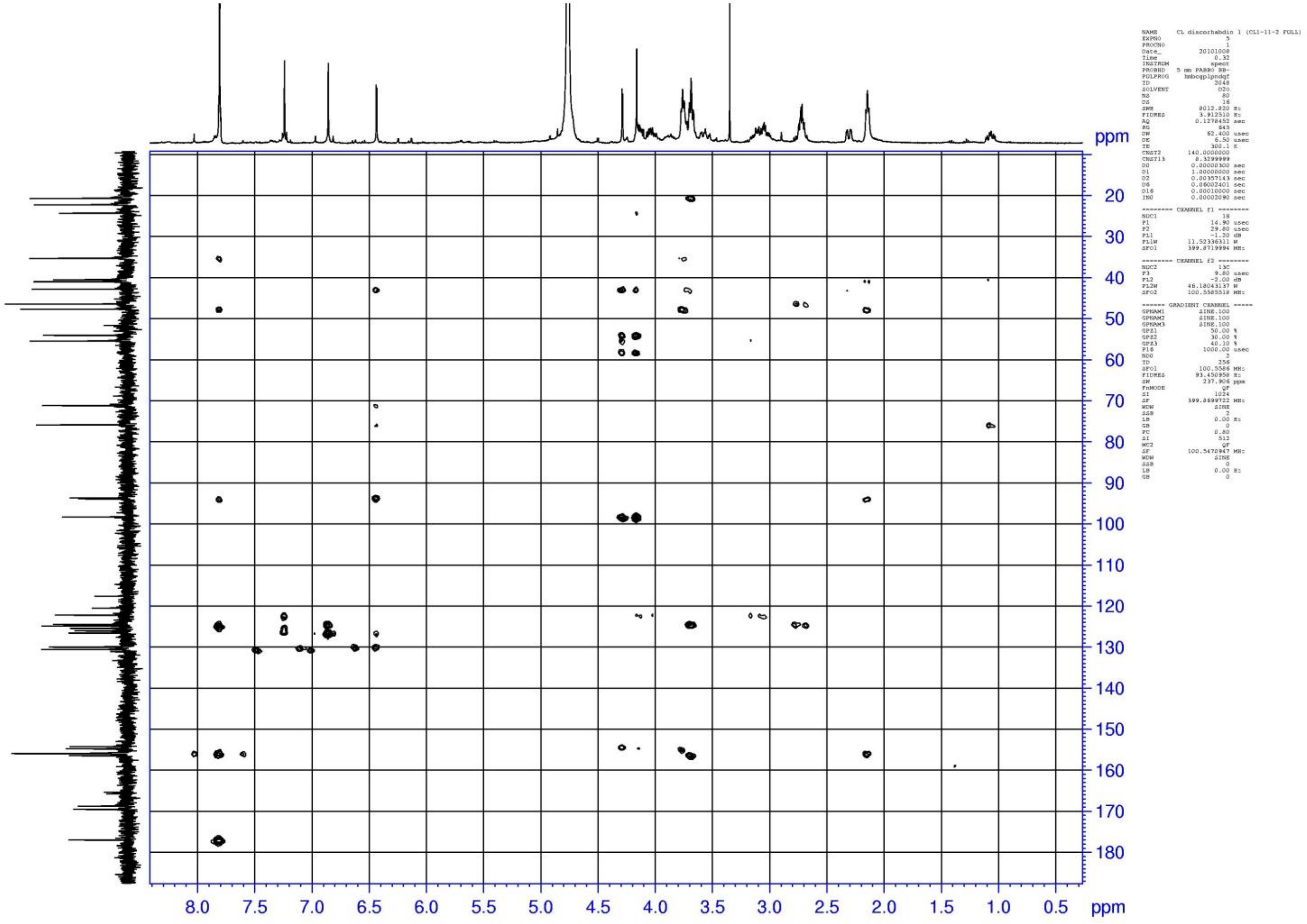

Figure S28. HMBC spectrum of compound **8** (TFA salt, D_2O, 400 MHz).

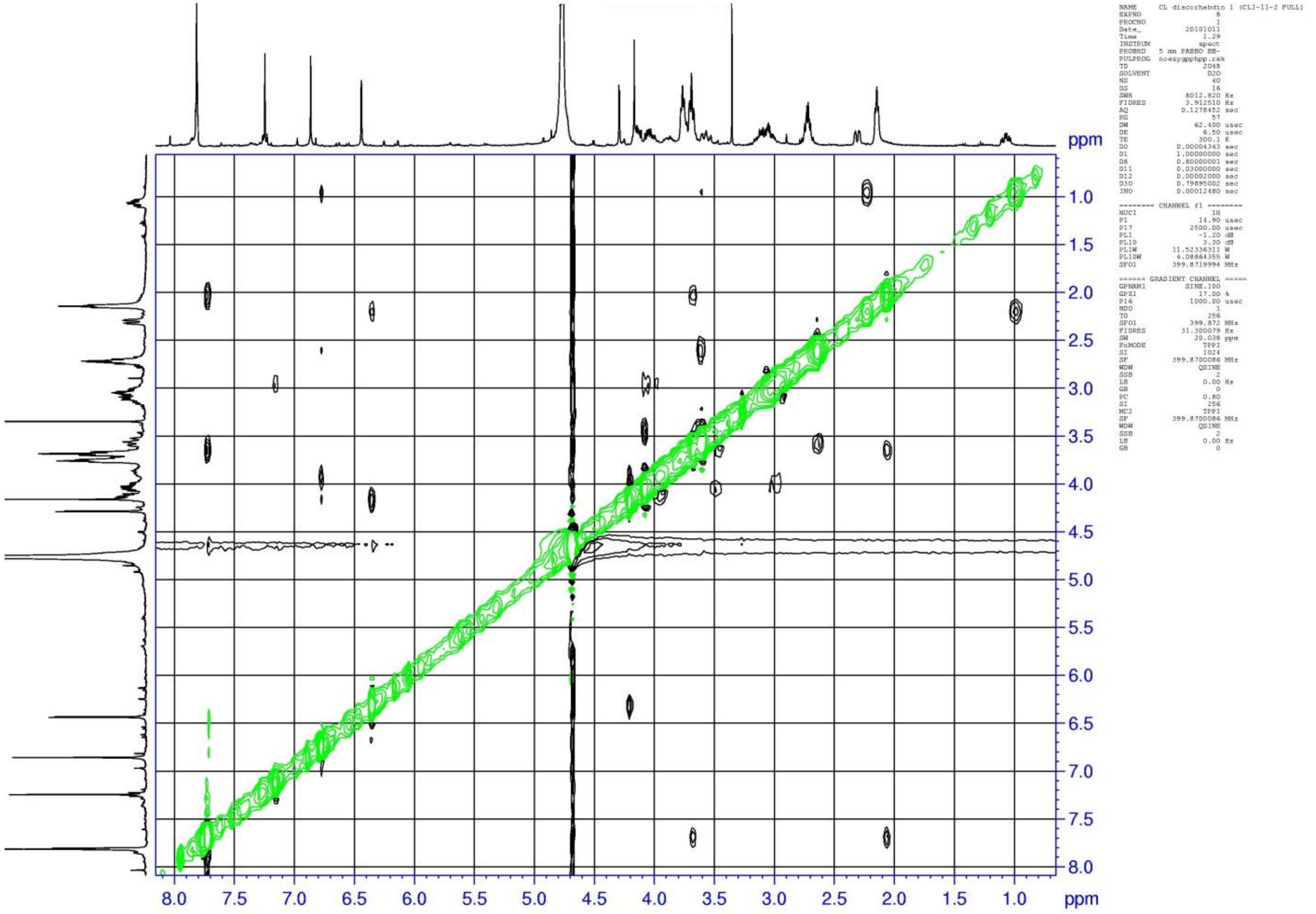

Figure S29. NOESY spectrum of compound **8** (600 ms mixing time, TFA salt, D_2O, 400 MHz).
